# Integrin inhibition differentially remodels vascular networks in lung metastases driven by vessel co-option or angiogenesis

**DOI:** 10.1007/s10456-026-10085-1

**Published:** 2026-08-20

**Authors:** Laura Marcos-Zazo, Iván Carrera-Aguado, Jesús Gómez-Escudero, Patricia Berlana-Galán, Irene Torre-Cea, Elena Guerra-Paes, Celia Redondo-Gonzalez, Juan Sánchez-Mateos, Daniel Cáceres-Calle, Óscar Maiques, Miguel Pericacho, Susana Fraile, Telmo Rodrigues-Teixeira, Carmen García-Macías, Omar García-Sánchez, Lorena Benito-Garzón, Fernando Sánchez-Juanes, José M. Muñoz-Félix

**Affiliations:** 1 Department of Biochemistry and Molecular Biology, University of Salamanca, Salamanca, Spain; 2 Institute for Biomedical Research of Salamanca (IBSAL), Salamanca, Spain; 3 Barts Cancer Institute, Queen Mary University of London, John Vane Science Building, Charterhouse Square, London, EC1M 6BQ UK; 4 Department of Physiology and Pharmacology, University of Salamanca, Salamanca, Spain; 5 Pathology Unit, Cancer Research Center (CIC-IBMCC), Salamanca, Spain; 6 Department of Human Anatomy and Histology, University of Salamanca, Salamanca, Spain

**Keywords:** Vessel co-option, Vascular promotion, Lung metastases, Angiogenesis, Cilengitide, Integrins

## Abstract

Tumor progression depends on an adequate blood supply to sustain oxygen and nutrients delivery. While tumor angiogenesis involves the formation of new blood vessels from pre-existing ones, vessel co-option represents a non-angiogenic vascularization strategy whereby tumor cells utilize pre-existing host vessels. Co-opted vessels have been considered refractory to anti-angiogenic therapies, and pharmacological modulation of co-opted vessels remains limited. In this study, we investigate the effects of low-dose cilengitide on tumor vascular remodeling in vessel co-option and angiogenic metastatic models. Our results reveal that cilengitide exerts distinct vascular effects depending on the mode of tumor vascularization. In vessel co-option-driven tumors, cilengitide treatment is associated with the remodeling of the co-opted vasculature into a more organized normalized vascular network, characterized by an increased number of functional blood vessels and enhanced vascular barrier integrity. In contrast, in angiogenic-driven tumors, cilengitide treatment promotes an expansion of the vascular network consistent with augmented, but structurally immature angiogenesis. Importantly, vascular remodeling in vessel co-option metastases is accompanied by enhanced blood vessel perfusion and reduced hypoxia, which correlates with enhanced responsiveness to chemotherapy. Conversely, in angiogenic metastases, the vascular network induced by low-dose cilengitide fails to support immunocompetent microenvironmental features and is associated with increased chemotherapy resistance. This study provides the first evidence that co-opted vasculature can be therapeutically targeted via integrin inhibition, suggesting vascular normalization remodeling as a potential strategy to overcome resistance in tumors undergoing vessel co-option.

**Supplementary Information:**

The online version contains supplementary material available at 10.1007/s10456-026-10085-1.

## Introduction

In order to maintain their growth, tumor cells need oxygen and nutrients, and these fuels are supplied by blood vessels. Due to the high proliferation rate of cancer cells, a broader vasculature is needed to support their increased oxygen demand. The main mechanism of vascularization in tumors is called angiogenesis, which consists in the formation of new blood vessels from pre-existing ones [1]. For this reason, anti-angiogenic therapy (AAT) was proposed and several strategies have been developed to target the most important players of tumor angiogenesis, such as vascular endothelial growth factor (VEGF), vascular endothelial growth factor receptor 2 (VEGFR2) or other tyrosine kinase receptors [2, 3].

Lately, the scientific community has focused on non-angiogenic tumors [4]. One of the vascularization mechanisms for these tumors is vessel co-option (VCO), in which cancer cells exploit pre-existing blood vessels instead of generating new ones [5–7]. This process is frequent in highly vascularized organs, such as lung, breast, liver or brain [4]. Unfortunately, VCO tumors are not responsive to AAT [5, 8–10]. Although some AAT compounds have displayed benefits in clinical practice (such as Bevacizumab), the majority of VCO tumors only show initial sensitivity to treatment, but after several weeks, they acquire an aggressive and invasive phenotype that promotes metastasis [1, 11]. This intrinsic resistance occurs because, in highly vascularized tissues, VCO tumors bypass the need for angiogenesis by coopting pre-existing host vasculature [4, 12]. Furthermore, AAT has shown other limitations, such as tumor hypoxia, inadequate tumor drug delivery or serious side effects [13, 14]. There is little information about the possible role of VCO in chemotherapy resistance or the induction of VCO after chemotherapy treatment. It is known that VCO creates an unfavorable tumor microenvironment (TME) for chemotherapy sensitivity [15]. Pichol-Thievend et al. demonstrated that chemoradiation treatment in glioblastoma cells induces a reprogramming process into a vessel co-opting and invasive cell state, which promotes cell infiltration into the brain tissue [16].

Other strategies have been designed for modifying tumor vasculature, such as vascular normalization or vascular promotion [17–19]. Vascular normalization is a therapeutic strategy that induces a transition from a chaotic and dysfunctional vascular network into a structurally and functionally normal vasculature, thereby enhancing treatment efficacy across a variety of diseases [20]. On the other hand, vascular promotion consists of inducing the formation of a fine vascular network by enhancing the quantity of tumor blood vessels and improving their structure and functionality. Consequently, this strategy reduces hypoxia and favors antitumoral therapies delivery [13, 14]. Both approaches are specially interesting in VCO tumors because, although their vessel density is higher, these blood vessels may not be functional as a result of the compression exerted by cancer cells [4, 21]. Different molecules have been tested to induce vascular promotion in preclinical models, for instance eribulin or lysophosphatidic acid (LPA), which have shown a reduction in tumor growth in combination with chemotherapy [22–25]. Nevertheless, the most relevant drug for vascular promotion is cilengitide, a cyclic RGD-mimetic peptide that inhibits α_v_β_3_ and α_v_β_5_ integrins. It was initially employed as AAT, but Reynolds et al. demonstrated that low-dose cilengitide (ldCil) has vascular promotion effects by upregulating VEGFR2 expression in endothelial cells (ECs) [26]. Since this outcome, ldCil has been employed in combination with different chemotherapies in preclinical angiogenic models, reducing tumor burden in pancreatic ductal adenocarcinoma and non-small cell lung cancer (NSCLC) [13, 27]. For this reason, we propose the employment of ldCil in order to modify VCO vasculature and overcome the resistance that VCO offers.

Our data show that in VCO-driven 4T1 metastases, although we expected a vascular pro-angiogenic phenotype, ldCil promotes the formation of a fine and stabilized vascular network through endothelial junction reinforcement and controlled vessel formation, resulting in a functional vasculature that enhances chemotherapy efficacy. In contrast, in angiogenic RENCA metastases, cilengitide exacerbates aberrant angiogenesis and vascular dysfunction. These findings identify a context-dependent effect of cilengitide on tumor vasculature and provide the first evidence of vascular remodelling with better outcomes in VCO tumors.

## Materials and methods

### Cell lines

The cancer cells 4T1 and RENCA were cultured in RPMI 1640 (Sigma-Aldrich) supplemented with 10% newborn calf serum (NCS), L-glutamine and 1% penicillin-streptomycin (Thermo Fisher Scientific) in Petri dishes. Human Umbilical Vein Endothelial Cells (HUVECs) were cultured in Endothelial Cell Growth Factor Medium 2 (EGM2) (Promo Cell) supplemented with SupplementMix for Endothelial Cell Growth Medium (Promo Cell) and 1% penicillin-streptomycin in Petri dishes. All cell lines were maintained at 37 °C in an atmosphere of 5% CO_2_ and checked for bacterial and mycoplasma contamination regularly.

### Cell viability assays

HUVECs cells were trypsinized, counted using a Neubauer counting chamber (Thermo Fisher Scientific) and seeded into a 96-well dish with a density of 10^3^ cells per well (100 µL/well). After 48 h growing at 37 °C and 5% CO_2_, the medium was discarded and 100 µL of the corresponding treatment were added: 2 µg/mL of cilengitide (Selleckchem) (high concentration) or 1 ng/mL of cilengitide (low concentration), in presence or absence of 100 ng/mL of VEGF (MedChem). All treatments were performed in EGM2 factors-free medium containing 0.5% Fetal Bovine Serum (FBS) (Thermo Fisher Scientific). Following a 48-hour treatment, medium was retired and 100 µL of Methyl Thiazolyl Tetrazolium (MTT) (Thermo Fisher Scientific) were added to each well. Cells were incubated with MTT at 37 °C and 5% CO_2_ for 4 h in darkness. Subsequently, 100 µL of 10% SDS-HCl were added to each well and cells were incubated 30 min at 37 °C and 5% CO_2_ before measuring the absorbance at 570 nm in a Thermo Scientific™ Multiskan™ FC Microplate Photometer.

The in vitro dose of cilengitide used in this study is comparable to the in vivo doses (50 µg/kg). Reynolds et al. reported the plasma concentration of cilengitide is approximately 1.7 nM ± 0.8 (equivalent to 1 ng/mL) and it induces the process of angiogenesis by upregulating VEGFR2 levels [26].

### Migration assay

HUVECs were cultured to assess their migration capacity in presence of 1 ng/mL cilengitide, 100 ng/mL VEGF or a combination of both treatments. For this assay, cells were seeded in 6 well-dishes until 95% confluence. At this moment, cells were exposed with the corresponding treatment and 15 min later, a wound was made in each well with a new 0.2-mL pipette tip. Images of the wells were taken at 0, 12 and 24 h post-wound using an Invitrogen™ EVOS™ XL Core Imaging System. Subsequently, images were analyzed using the Wound Healing Size Tool plugin from Fiji/ImageJ software (1.54f version on Windows) in order to obtain the wound area percentage.

### Spheroids assay

20 µL drops of 0.25% Methylcellulose (Sigma-Aldrich) in EGM2 containing 750 HUVECs cells were seeded to form spheroids by hanging drop method on the underside of a 10 cm Petri dish lid. The lid was then inverted over the dish, filled with Phosphate Buffered Saline (PBS). 24 h after incubation at 37 °C, drops were carefully collected with a 1 mL-pipette tip, and mixed 1:1 with a pH-adjusted solution of collagen I to a final concentration of 1 mg/mL. 250 µL of the mix was seeded in a well of a 24-well dish. After 1 h of incubation at 37 °C, 500 µL EGM2 with 0.5% FBS and 100 ng/mL of VEGF were added. 4 h after VEGF addition, cilengitide was added at a concentration of 1 ng/mL exclusively to the spheroids in this experimental condition. The day after, the spheroids were imaged using a Invitrogen™ EVOS™ XL Core Imaging System, and total protrusion length was quantified using Fiji/ImageJ software (1.54f version on Windows).

### Endothelial cell junction assay

10^4^ HUVECs were seeded on gelatin-coated µ-Slide 15 Well 3D ibidi slides (IBIDI) and cultured until they reached confluence. Cells were serum-starved for 12 h by culturing them in EGM2 without growth factors and supplemented with 0.5% FBS. After starvation, they were treated with 1 ng/mL of cilengitide in presence or absence of 100 ng/mL VEGF in EGM2 with 0.5% FBS for 24 h. Cells were fixed in 3.7% p-formaldehyde (PFA) (Casa Álvarez) for 15 min, permeabilized in 0.1% Triton X-100 for 10 min, and blocked in Bovine Serum Albumin (BSA) (Sigma-Aldrich) 2% for another 30 min.

The primary antibody anti-VE-cadherin (Thermo Fisher Scientific Cat# MA5-28541, RRID: AB_2745500) or anti-CD31 (Thermo Fisher Scientific Cat# MA5-37858, RRID: AB_2897778) was diluted in blocking solution (2% BSA and 0.3% Triton X-100 in PBS) at 1:100, and incubated for 24 h at 4 °C. After 3 washes with PBS, the secondary goat antibody anti-rat Alexa Fluor 568 (Sigma-Aldrich Cat# SAB4600085, RRID: AB_3492074) or the secondary goat antibody anti-rabbit Alexa Fluor 488 (Thermo Fisher Scientific Cat# A-11034, RRID: AB_2576217) was diluted 1:250 in blocking solution (2% BSA and 0.3% Triton X-100 in PBS) and cells were incubated overnight at 4 °C. The next day, they were washed with PBS 3 times, and mounted with ProLong™ antifade reagent (Thermo Fisher Scientific), which contains DAPI, a nuclear counterstain [28]. Images of VE-cadherin were acquired using a Zeiss LSM 510 confocal microscope and CD31 using a Zeiss Axioplan fluorescence microscope. Types of junctions were classified as linear, reticular or intermittent depending on their pattern [29], and the proportion of each of them was calculated for every image. All these analyses were done blindly by 6 blind reviewers.

### In vitro permeability assay

HUVECs were grown in the upper chambers of 6-well Transwell inserts (0.4 μm pore size; Corning, NY, USA) until a confluent monolayer was formed. For endothelial permeability assays, HUVECs were starved in serum-free EGM2 for 3 h. After starvation, the upper and lower chambers were treated with 1 ng/mL of cilengitide in presence or absence of 100 ng/mL VEGF in EGM2 with 0.5% FBS for 16 h in EGM2 supplemented with 0.5% FBS. Subsequently, fluorescein isothiocyanate (FITC)-labeled dextran 40 (Sigma-Aldrich) at a 1 ng/mL concentration in basal medium was added as a tracer to the upper chamber, and cells were incubated at 37 °C. At 6 h, 100 µL of the medium in the lower chamber were collected into a black 96-well dish. Fluorescence was measured with a Varioskan LUX Multimode Microplate Reader at 485 nm excitation and 530 nm emission wavelengths. Fluorescence intensity was employed to determine the permeability of the ECs monolayer: a higher fluorescence indicated higher permeability.

### Western blot

Western blot was carried out to confirm the expression of β_1_, β_3_ and β_5_ integrins in 4T1, RENCA and HUVECs cell lines. Cells were cultured as described above, trypsinized and seeded at 200,000 cells/well in 6-well dishes. When cells were 90% confluent, the RIPA lysis buffer (Thermo Fisher Scientific) was applied to obtain proteins. Cell lysates were centrifuged at 14,000 g for 20 min at 4 °C, supernatants were recovered, and protein quantification was performed using the Pierce™ BCA Protein Assay Kit (Thermo Fisher Scientific). Samples were prepared with 15 µg of protein and loaded onto 10% polyacrylamide gels to carry the electrophoretic separation. Subsequently, proteins were transferred to nitrocellulose membranes (Amersham) by electroblotting. Afterwards the blocking solution 3% BSA in Tris-Buffered Saline with Tween-20 (TTBS) was applied for 1 h at room temperature, before an overnight incubation at 4 °C with the corresponding primary antibody (Supplementary Table 1) at 1:500 dilution. The following day, membranes were incubated during 2 h at room temperature in darkness with the secondary antibody goat anti-IgG StarBright™ Blue 520 at 1:500 dilution. β-actin was used as loading control. Membranes were incubated with the actin-rhodamin antibody (Bio-Rad). Finally, the protein visualization was carried out using a ChemiDoc Imaging System (Bio-Rad).

### Cell immunofluorescence

Immunocytochemistry was performed with RENCA, 4T1 and HUVECs cell lines in order to check the expression of β_1_, β_3_ and β_5_ integrins. 2·10^3^ cells/well were seeded in a 2-well Chamber Slide (Thermo Fisher Scientific) and incubated for 24 h at 37 °C. After the incubation, cells were washed with PBS, fixed with 3.7% PFA and washed again with PBS. Subsequently, cells were permeabilized by incubating for 10 min with 0.1% Triton X-100 and washed with PBS. Then, the blocking solution (2% BSA and 0.3% Triton X-100) was applied for 10 min. Next, 150 µL of a 1:150 dilution of the corresponding primary antibody (Supplementary Table 1) and 150 µL of Acti-stain™ 488 phalloidin (Santa Cruz Biotechnology Cat# sc-363791, RRID: AB_2631056) were added and incubated in darkness overnight at 4 °C. The next day, the samples were washed with PBS in agitation, before washing with 0.05% Tween-20 for 10 min. Finally, the secondary antibody goat anti-mouse AlexaFluor 568 (Thermo Fisher Scientific Cat# A-21124, RRID: AB_2535766) were applied at a 1:150 dilution for 45 min at room temperature. Then, cells were washed again with PBS, the chamber slides were retired and the samples were mounted with the solution ProLong™ antifade reagent. Images of the samples were taken with Leica DM4 B Optical Microscope.

### In vivo models of pulmonary metastases

The breast cancer cell line 4T1 was used to generate VCO lung metastases, whereas the renal carcinoma cell line RENCA was employed to generate angiogenic lung metastases [5, 10]. We injected 2·10^5^ 4T1 or RENCA cells intravenously into the tail vein of 6-week old BALB/c females (Charles River), following the protocol described by Cuypers et al. [30]. These models were chosen due to their extensive characterization in previous studies, serving as well-established experimental platforms for studying VCO and angiogenic tumor growth, respectively [5, 10, 30].

In the 4T1 model, mice were split into two treatment groups: placebo (*n* = 5) and ldCil (*n* = 5). Placebo mice were treated intraperitoneally with 100 µL PBS and mice from the ldCil group were treated intraperitoneally with 50 µg/kg cilengitide diluted in PBS at days 4, 7 and 10 post-injection. Mice were sacrificed 13 days after intravenous injection by cervical dislocation. In the RENCA model, mice were divided into the same treatment groups (*n* = 5 placebo and *n* = 4 ldCil) and were administered the same doses of PBS and cilengitide at days 9, 12, 17 and 20 post-injection. Animals were sacrificed 27 days after intravenous injection by cervical dislocation. The cilengitide dose was chosen based on published research by Wong et al., where authors identified that the in vivo doses for vascular promotion therapies is 50 µg/kg intraperitoneal [13]. These authors selected this dose based on previous studies [26].

An in vivo chemotherapy study was carried out to evaluate the benefits of chemotherapy when using ldCil. In the 4T1 model, 6-week old BALB/c female mice were treated with vehicle (corn oil, *n* = 8) (Sigma-Aldrich), 5 mg/kg paclitaxel (Ptax^5^) (MedChem) diluted in corn oil (*n* = 8) and Ptax^5^ in combination with 50 µg/kg cilengitide (*n* = 9*)* at days 4, 7 and 10 post-injection. In the RENCA model, 6-week old BALB/c female mice were treated with vehicle (corn oil, *n* = 5), Ptax^5^ diluted in corn oil (*n* = 5) and Ptax^5^ in combination with 50 µg/kg cilengitide (*n* = 4) at days 9, 12, 17 and 20 post- injection. Mice were sacrificed on days 13 and 27, respectively. Another chemotherapy experiment was carried out using carboplatin, in combination or not with ldCil, in the 4T1 lung metastasis model to determine whether the therapeutic effect observed previously could be reproduced in this model using a different chemotherapeutic agent (Supplementary material and methods).

An in vivo AAT experiment was carried out to evaluate the possible effects of the VEGF/VEGFR2 inhibitor sorafenib in the vascular effects induced ldCil in 4T1 and RENCA models (Supplementary material and methods).

All procedures involving live animals were performed by qualified technicians and approved by the Bioethics Committee of the University of Salamanca, as well as the Regional Ministry of Agriculture, Livestock and Rural Development of Castilla y León. These processes complied with the current regulations for animal experimentation established in the Declaration of Helsinki (Law 15/2007, of July 3).

### Lung tissue processing

After the mice were sacrificed, lungs were taken and processed for histology. Lungs were inflated with 3.7% PFA and submerged in 3.7% PFA in Falcon™ tubes. Then, lungs were transferred to ethanol 70% (Casa Álvarez), divided into 5 lobes and embedded in paraffin [31]. Finally, 3 μm sections containing the 5 lung lobes were obtained in glass slides to perform each of the stainings described below.

### Haematoxylin-eosin staining

Haematoxylin-eosin (H&E) staining was performed for morphological analysis of the metastases. Lung tissue samples were deparaffinized with Clear SX85 (Casa Álvarez) before rehydration with progressively lower concentrations of ethanol (100%, 80%, 70% and 50%). Afterwards, samples were washed out with distilled water and immersed in Gill’s hematoxylin (Casa Álvarez) until tissues dyed. The excess dye was removed with distilled water, and samples were stained with 1% eosin (Casa Álvarez). Then, samples were rinsed with water, underwent dehydration by immersion in a gradient of ethanol (80% and 100%) and were washed with Clear SX85. Finally, the slides were mounted with glass coverslips and a drop of DPX mounting medium (Casa Álvarez). Images of the slides were taken with Olympus BX51 Optical Microscope and Leica DM4 B Optical Microscope for the analysis of number of metastases, tumor area, mean tumor size and perimeter-area ratio.

### Immunofluorescence

We carried out several immunofluorescence stainings: endomucin (endothelial marker)/podoplanin (pneumocyte marker) [30, 32], endomucin/PDGFRβ (Platelet-Derived Growth Factor Receptor Beta) (pericyte marker) [32, 33], endomucin/desmin (pericyte marker) [33] and endomucin/Ki67 (cell proliferation marker) [34].

Tissue deparaffinization and rehydration were performed in the same way as in the H&E staining and samples washed with distilled water and PBS. Afterwards, antigen retrieval was carried out by immersing the samples in the corresponding solution (Supplementary Table 2). Next, a 1 h-incubation, with the blocking solution BSA 3% and 0.3% Triton X-100 in PBS was performed. Subsequently, samples were incubated overnight at 4 °C with 30 µL of a 1:100 dilution with the corresponding primary antibody (Supplementary Table 3). The next day, the primary antibody was removed by washing with PBS and samples were incubated in darkness for 1 h with 30 µL of a 1:200 dilution of the corresponding secondary antibody (Supplementary Table 3). After the incubation period, samples were washed with PBS and distilled water. Finally, the slides were mounted with the mounting solution ProLong™ antifade reagent, which contains DAPI. Images of the samples were taken with a Leica DM4 B Optical Microscope and the Leica Application Suite X (LAS X) 3.8 software for blood vessels analysis and pericyte coverage.

### β_3_ and β_5_ integrins immunohistochemistry with Monosan Plus kit in lung tissue

β_3_ and β_5_ integrins immunohistochemistry were performed using the commercial kit Monosan Plus (AP) Broad Spectrum (Fast Red) (MONOSAN) to analyze the protein distribution in placebo lung metastases. Tissue deparaffinization and rehydration were performed in the same way as in the H&E staining and samples washed with distilled water and PBS. Subsequently, antigen retrieval was carried out (Supplementary Table 2) and the commercial kit protocol was applied using 1:100 dilutions of anti-β_3_ and anti-β_5_ integrin primary antibodies (Supplementary Table 1).

### Immunohistochemistry with NeoStain ABC kit

CD8, Arginase-1 (M2 macrophages marker) [35], FOXP3, granzyme B and CD11b (myeloid cells marker) [36] immunohistochemistry was carried out for tumor immune microenvironment (TIME) analysis; and glucose transporter 1 (GLUT-1) and carbonic anhydrase IX (CAIX) immunohistochemistry were performed for tumor hypoxia analysis [37, 38].

Tissue samples were deparaffinized and rehydrated in the same way as in the H&E staining and washed with distilled water and PBS. Afterwards, antigen retrieval was carried out by immersing the samples in the corresponding solution (Supplementary Table 2). Subsequently, samples were washed in agitation with PBS and treated with a 3% H_2_O_2_ solution. Then, the blocking solution (3% BSA and 0.3% Triton X-100) was applied for 1 h. After removing the remaining solution, 30 µL of the corresponding primary antibody (Supplementary Table 1) were applied at a 1:100 dilution and tissues were incubated overnight at 4 °C. The next day, primary antibodies were washed with PBS and the biotinylated secondary antibody of the commercial kit NeoStain ABC, Horseradish Peroxidase (HRP) Detection Bulk Kit for Mouse and Rabbit Antibodies (NeoBiotech) was added for 20 min. After the incubation period, the remaining antibody was washed with PBS before applying the streptavidin-HRP complex from the kit for 20 min. The strong affinity between streptavidin and biotin facilitates the formation of the biotin-streptavidin-HRP complex. Afterwards, the samples were washed with PBS before applying diaminobenzidine (DAB) (Merck Millipore) for 10 min, which was transformed by HRP in an insoluble brown precipitate, staining the antigens. Then, the samples were rinsed with water and immersed in Mayer’s haematoxylin (Casa Álvarez) for 5 min. After that, tissues were washed again with water and PBS and dehydrated in a gradient of ethanol (80%, 100%) and Clear SX85. Finally, samples were mounted with glass coverslips and a drop of DPX mounting medium. Images of the samples were taken with an Olympus BX51 Optical Microscope and a Leica DM4 B Optical Microscope.

### Number of metastases, tumor area, mean tumor size and perimeter/area ratio

H&E-stained sections were analysed using Fiji/ImageJ software (version 1.54f, Windows). For each animal, all available lung lobes were sectioned and analysed. Tumor number was determined by counting all metastatic lesions across the analysed sections. Total tumor area was obtained by manually delineating each tumor lesion. Mean tumor size was calculated as the total tumor area divided by the number of tumors, representing the average lesion area per section. Perimeter/area ratio was calculated after manually delineating each tumor lesion to obtain its perimeter and area measurements. The resulting perimeter-to-area ratio provided an estimate of lesion morphology, with lower values corresponding to more rounded lesions and higher values associated with more irregular or invasive lesions. For each parameter, the mean of each mouse was calculated. Measurements were performed in pixels^2^.

### Blood vessel density and coverage analysis

Immunofluorescence images of endomucin/PDGFRβ and endomucin/desmin stainings were analyzed with Fiji/ImageJ software (1.54f version on Windows). For each mouse, seven tumors were randomly selected to study blood vessel density and pericyte coverage. Blood vessel density was determined by counting individual vessels (demonstrated by endomucin positive areas [32]) and dividing this number by the tumor area (expressed in pixels^2^), which was calculated by delineating the tumor shape in Fiji/ImageJ software (1.54f version on Windows). On the other hand, pericyte coverage was analyzed by quantifying the number of individual blood vessels and determining if each vessel was closely associated with a pericyte, demonstrated by the presence of PDGFRβ or desmin [33]. The percentage of pericyte coverage was expressed as the number of blood vessels associated with pericytes relative to the total number of blood vessels. For both parameters, the mean of each mouse was calculated.

### Microvascular growth pattern analysis and epithelial loss area analysis

The microvascular growth pattern in tumors was determined from the double immunofluorescence of endomucin and podoplanin. Taking into account previous findings by Bridgeman et al. and Szabo et al., tumors with the typical honeycomb structure, demonstrated by podoplanin preservation and epithelial cells being in contact to cancer cells, were considered as alveolar-interstitial histological growth pattern (HGP) (VCO pattern), whereas tumors without podoplanin were tagged as pushing HGP (angiogenic pattern) [5, 39]. For each animal, all of the tumors were analysed.

Moreover, using the Threshold tool in Fiji/ImageJ software (1.54f version on Windows) we quantified tumor areas without podoplanin and divided the result by the total tumor area, which was determined by delineating tumor shape in Fiji/ImageJ software (1.54f version on Windows). The resulting parameter was named as *Epithelial Loss Area*. Area measurements were calculated in pixels^2^. Seven tumors per mouse were randomly selected to perform the analysis. The mean value per mouse was calculated.

### Tumor hypoxia analysis

For tumor hypoxia evaluation, GLUT-1 and CAIX stainings were analysed. We randomly selected seven tumors per mouse to perform the study. Images were analyzed using Fiji/ImageJ software (1.54f version on Windows) and hypoxia was calculated as GLUT-1 or CAIX positive areas/tumor area. Tumor areas were manually delineated and the Color Threshold tool was applied to selectively measure brown-stained areas corresponding to GLUT-1 or CAIX areas. Identical threshold settings were applied to all images. Area measurements were calculated in pixels^2^.

### TIME analysis

For TIME analysis tumor T CD8^+^ lymphocytes infiltration, T_reg_ lymphocytes infiltration, CD11b positive cells infiltration, M2 macrophages infiltration and granzyme B secretion were taken into account. Images of the different stainings were analyzed using Fiji/ImageJ software (1.54f version on Windows). We randomly selected seven tumors per mouse to perform the analysis. T CD8^+^ cells infiltration was calculated from the number of T CD8^+^ lymphocytes, dividing them into peripheral and intratumoral cells. T_reg_ lymphocytes infiltration was calculated as the number of cells per tumor. CD11b positive cells and M2 macrophages infiltration was obtained as the number of CD11b^+^ or M2 macrophages per tumor area in pixels^2^. Granzyme B was calculated as DAB positive areas per tumor area. Images of stained tumor sections were analysed delineating tumor area and using the Color Threshold tool to selectively identify and segment the DAB-positive brown staining corresponding to granzyme B. Uniform threshold parameters were applied to all images. Area measurements were calculated in pixels^2^. For each parameter, the mean value per mouse was calculated.

### Blood vessel perfusion and pimonidazole analysis

Another in vivo experiment was performed to evaluate vessel perfusion and tumor hypoxia through the detection of the hypoxia marker pimonidazole after ldCil treatment. In the 4T1 model mice were treated with vehicle (*n* = 6) or 50 µg/kg cilengitide (*n* = 6) at days 4, 7 and 10 post-injection of 4T1 tumor cells. In the RENCA model mice were treated with vehicle (*n* = 6) or 50 µg/kg cilengitide (*n* = 6*)* at days 9, 12, 17 and 20 post-injection of the RENCA cell line. Mice were sacrificed by cervical dislocation on days 13 and 27, respectively.

At the end point of the experiments, 1 h before the sacrifice 100 µL of pimonidazole-HCl (Hypoxyprobe™ kit) (Hypoxyprobe) (60 mg/kg) were intravenously administered and 15 min before the sacrifice 100 µL of 2000 kDa FITC-dextran (Sigma-Aldrich) (10 mg/kg) were injected intravenously [40, 41]. Immediately after sacrifice, lungs were perfused with PBS and 3.7% PFA via intracardiac injection. Afterwards, lungs were harvested and sequentially cryopreserved in sucrose 10%, sucrose 20%, sucrose 30%. Then, lungs were embedded in OCT (Optimal Cutting Temperature) compound and subsequently frozen. Finally, 3 μm sections were obtained in the cryostat.

For blood vessel perfusion analysis, tissue sections were mounted using Prolong™ antifade with DAPI. All of the tumors were imaged using a Leica DM 4B microscope to perform the evaluation. FITC-dextran fluorescence was used to identify perfused blood vessels. Using Fiji/ImageJ software (1.54f version on Windows), we determined the number of perfused blood vessels per tumor area, which was calculated by manually delineating each metastasis. Area measurements were performed in pixels^2^.

For pimonidazole analysis, frozen tissue sections were fixed in acetone at − 20 °C for 20 min, washed with PBS, and incubated in blocking solution (3% BSA and 0.3% Triton X-100) for 1 h. Then samples were incubated with a 1:100 dilution of the primary antibody anti-pimonidazol (Hypoxyprobe™ Kit) (Hypoxyprobe). The rationale of this kit is based on the conversion of pimonidazole-HCl into pimonidazole in hypoxic cells. The following day, the primary antibody was removed by washing with PBS and samples were incubated in darkness for 1 h with a 1:200 dilution of the secondary antibody Sigma-Aldrich Cat# SAB4600085, RRID: AB_3492074. Finally, samples were mounted with Prolong™ antifade with DAPI. Images of the samples were taken using a Leica DM4 B Optical Microscope and seven tumors per mouse were analysed to evaluate tumor hypoxia. Given that pimonidazole requires functional blood flow to reach the tissue, pimonidazole stained area was normalized according to the number of perfused vessels. Using Fiji/ImageJ software (1.54f version on Windows) we determined the hypoxic area (in pixels^2^) per number of perfused blood vessels. The Color Threshold tool was applied to selectively measure pimonidazole areas and the number of perfused blood vessels was determined by counting individual vessels (demonstrated by FITC-dextran fluorescence).

### Statistical analysis

Data were analyzed using GraphPad Prism 8.0.1. software. Comparison between two groups with normally distributed values were analyzed using the Student’s *t* test, whereas the non-parametric Mann-Whitney *U* test was applied to analyze percentages results and ratios. Kruskal Wallis test was used to compare the means between three or more groups with percentages results, followed by the multiple comparisons test Dunn’s. Comparisons between three or more groups with normally distributed values were analyzed using one-way ANOVA test, followed by the multiple comparisons test Sidak. Two-way ANOVA and post hoc Tukey´s test were used in wound healing assays, kind of cell-cell junction in HUVECs and viability assays with chemotherapy. Fisher’s exact test was employed to determine changes in T CD8^+^ lymphocytes distribution within tumors and changes in the HGP of the metastasis. Outliers were checked using Grubbs’ test. A *p*-value equal to or lower than 0.05 was considered statistically significant.

## Results

### β_3_, β_5_ and β_1_ integrins expression in VCO and angiogenic lung metastases supports susceptibility to cilengitide treatment

**Fig. 1:**
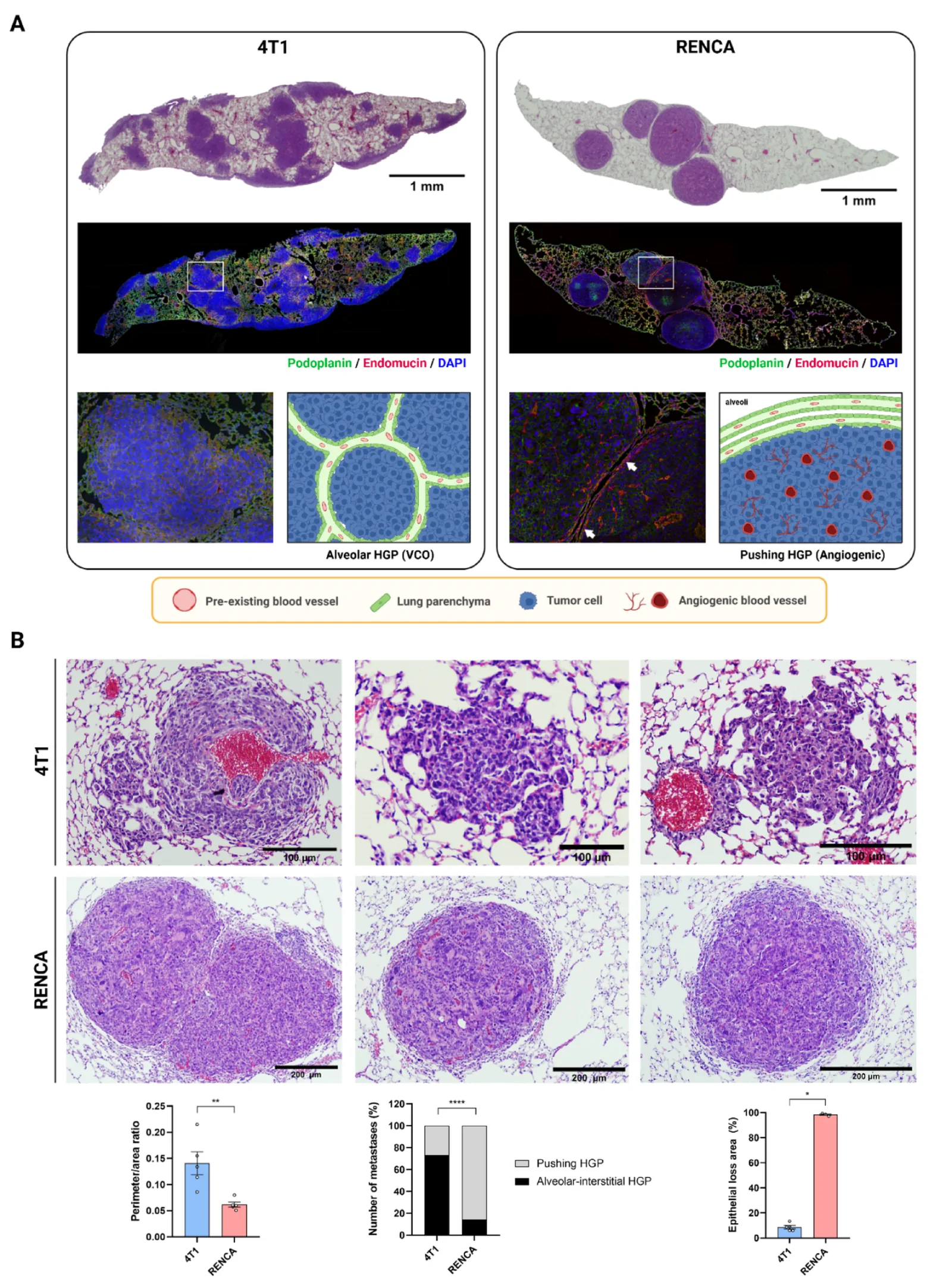
Morphological features of 4T1 and RENCA lung metastases. **A** *H&E staining and podoplanin/endomucin double immunofluorescence in one lung lobe bearing 4T1 (left panel) or RENCA (right panel) lung metastases.* H&E staining shows the morphology of the tumors and the tumor cells distribution along the lung parenchyma. In VCO tumors, cells disseminate through the lung occupying the air spaces and the lung walls. In contrast, the angiogenic metastases of the RENCA model grow filling the alveoli and compressing lung parenchyma displaying a *pushing* HGP (indicated by white arrows in the picture) with regular spheroid morphology. Double immunofluorescence of podoplanin (pneumocyte marker) and endomucin (endothelial marker) shows lung parenchyma structure (green) and vasculature (red), demonstrating podoplanin preservation in the 4T1 model and podoplanin absence in the RENCA model. Blood vessels of 4T1 tumors are aligned with the alveolar epithelial cells layer, demonstrating VCO growth, while the blood vessels of the RENCA model are in the core of the lesion isolated from the lung parenchyma, suggesting neo-angiogenic vessel formation. Nuclei tumor cells are shown in blue (DAPI). Scale bar = 1 mm. The background of the H&E staining lobe images has been removed using PowerPoint in order to clarify the observation of the lung metastases. Schemes were created with BioRender.com (license: QB29VG0TJ8). **B** *Perimeter/area ratio*,* distribution of HGP and* *Epithelial Loss Area* *analysis in 4T1 and RENCA lung metastases.* The perimeter/area ratio of the metastases was calculated for both experimental models (*n* = 5 in 4T1 and *n* = 4 in RENCA). 4T1 tumors have a significantly higher perimeter/area ratio than RENCA metastases, indicating more complexity due to their irregular shape, responsible for its higher perimeter. HGP have been classified according to the presence or absence of the epithelial marker podoplanin. Moreover, we determined the *Epithelial Loss Area* percentage in both experimental models (*n* = 5 in 4T1 and *n* = 3 in RENCA). RENCA metastases show a significantly higher *Epithelial Loss Area* than 4T1 ones, demonstrating angiogenic and VCO growth respectively. Individual values are represented in the graph and the bars represent the mean ± SEM. Scale bar = 100–200 μm. * *p* ≤ 0.05; ** *p* ≤ 0.01; **** *p* ≤ 0.0001. Mann-Whitney *U* test (perimeter/area ratio and *Epithelial Loss Area*) and Fisher’s exact test (HGP distribution)

In this study, we utilized two well-established murine models of lung metastasis to investigate the distinct vascularization strategies. Specifically, we employed the intravenous injection of 4T1 cells to study VCO and the intravenous injection of RENCA cells to model tumor-induced angiogenesis. These represent the most robust and best-characterized experimental murine models for their respective vascular patterns, and our results faithfully reproduced these phenotypes. According to previous studies, 4T1 lung metastases develop VCO growth [5, 10], showing alveolar HGP, in which tumor cells fill the air spaces in the lung; and interstitial HGP, where cells spread along lung walls [5, 12] (Fig. 1A). In contrast, RENCA cells undergo neoangiogenesis, following a *pushing* HGP, in which tumor cells grow inside the alveoli and compress the lung parenchyma to expand tumor growth [5, 10] (Fig. 1A). We confirmed these HGP by analyzing the perimeter/area ratio of the tumors in both experimental models, demonstrating that a higher perimeter/area ratio is associated with VCO (Fig. 1B). Moreover, we compared the number of metastases with each HGP (alveolar-interstitial or *pushing*). We observed that about 70% of 4T1 lung metastases were alveolar-interstitial, in contrast to RENCA lung metastases, of which approximately 80% exhibited *pushing* HGP (Fig. 1B). Since lung metastases undergoing VCO preserve the lung parenchyma, whereas those with a *pushing* HGP do not grow along the lung epithelium but rather displace the alveoli, we calculated the tumor area where the epithelial marker podoplanin was absent as *Epithelial Loss Area*. We observed that *Epithelial Loss Area* was very low in 4T1 lung metastases opposite to in RENCA lung metastases (more than 95%) (Fig. 1B).

**Fig. 2:**
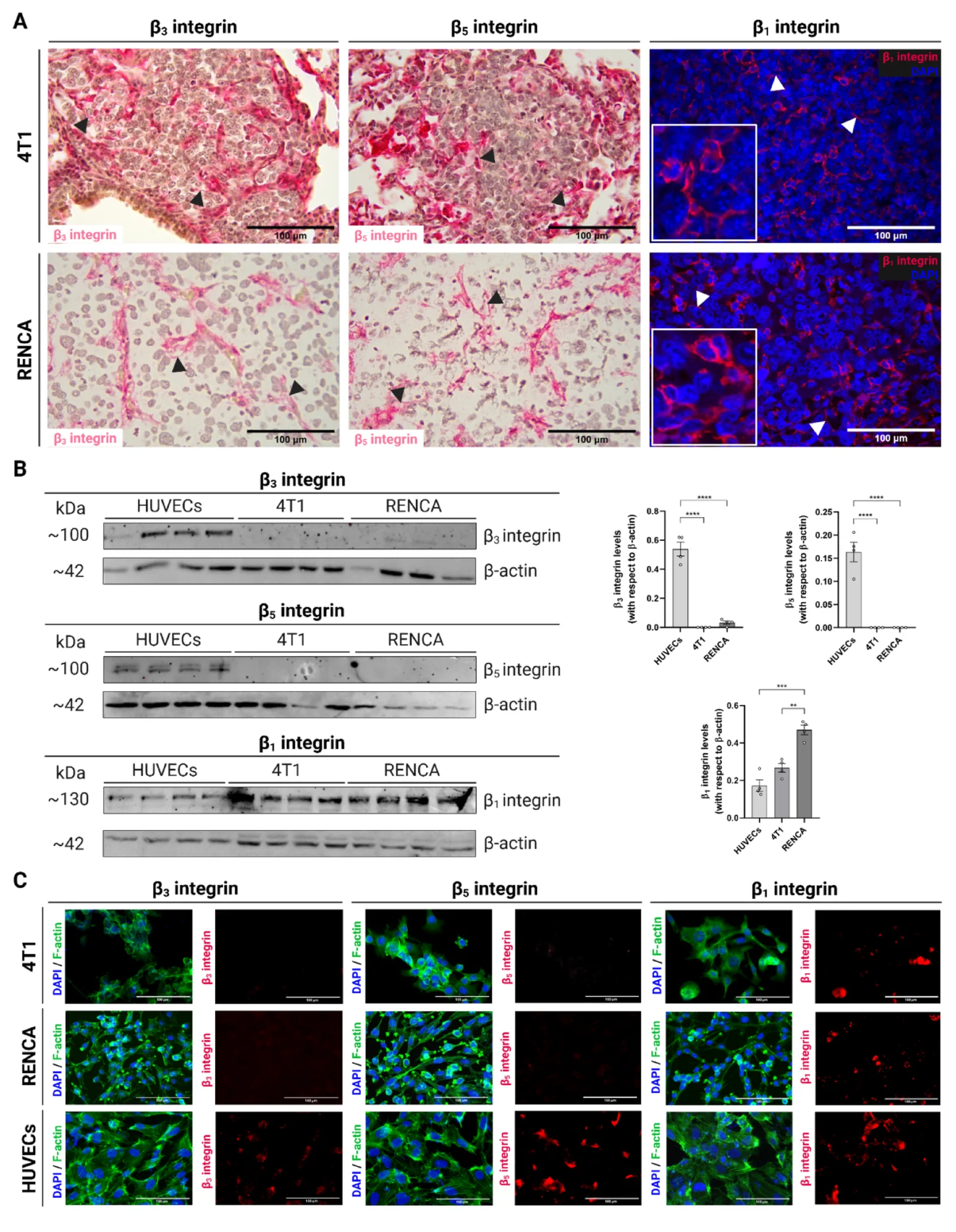
β_3_, β_5_ and β_1_ integrin levels and localization in 4T1 and RENCA *in vivo* models and in 4T1, RENCA and HUVECs cell lines. **A** *β*_*3*_, *β*_*5*_ *and β*_*1*_ *integrin immunostaining of 4T1 and RENCA lung metastases.* β_3_ integrin and β_5_ integrin are distributed among tumor cells in the extracellular space surrounding tumor cells forming vessel-like structures. β_1_ integrin is expressed predominantly in 4T1 and RENCA tumor cells *in vivo*, and also in the extracellular space around tumor cells forming vessel-like structures. Black and white arrows indicate these vessel-like structures and squares represent the areas that have been magnified below. Scale bar = 100 μm. **B** *Western blot analysis of β*_*3*_, *β*_*5*_ *and β*_*1*_ *integrins in HUVECs*,* 4T1 and RENCA cells.* The left panel shows western blot membranes for integrins and β-actin (loading control) detection. The right panel includes data analysis of β_3_, β_5_ and β_1_ integrins levels with respect to β-actin in each cell line (*n* = 4). Individual values are represented in the graph and the bars represent the mean ± SEM. β_3_ integrin is mainly expressed in HUVECs and also slightly in the RENCA cell line, whereas no expression is observed in 4T1 cells. β_5_ integrin only appears in HUVECs and is totally absent in 4T1 and RENCA cell lines. β_1_ integrin is expressed across all three cell lines. **C** *Cell immunofluorescence of β*_*3*_, *β*_*5*_ *and β*_*1*_ *integrins in 4T1*,* RENCA and HUVECs cell lines.* While β_1_ integrin staining shows levels of this protein across all three cell lines, β_3_ and β_5_ integrins appear mainly in HUVECs. **p ≤ 0.01; *** *p* ≤ 0.001; **** *p* ≤ 0.0001. One-way ANOVA, post hoc Sidak’s test (**B**)

As cilengitide shows high affinity for α_v_β_3_ integrin (IC_50_ = 0.61 nM), α_v_β_5_ integrin (IC_50_ = 8.4 nM) and also α_5_β_1_ integrin (IC_50_ = 14.9 nM) [42], we checked the expression of these proteins in lung tissue in our 4T1 and RENCA experimental models (Fig. 2A). Integrin expression analysis in 4T1 and RENCA models showed β_3_, β_5_ and β_1_ integrin positive areas with vessel-like structures among cancer cells in PBS-treated metastases of both experimental models. Moreover, in the case of β_1_ integrin, we observed expression in 4T1 and RENCA cells (Fig. 2A). Immunocytochemistry and western blot analysis of 4T1, RENCA and HUVECs cell lines confirmed that β_1_ integrin was expressed in the three cell lines, whereas β_3_ integrin appeared mainly in HUVECs and also slightly in RENCA cells, and β_5_ integrin was only expressed in ECs (Fig. 2B and C). To our knowledge, β_3_ and β_5_ integrins are expressed in ECs across different tumor models, specifically in angiogenic blood vessels [43, 44]. In fact, these integrins have been explored as key mediators in tumor angiogenesis and their expression in blood vessels indicates activated ECs [13, 45]. Although there is no information about the expression and function of these proteins in co-opted blood vessels and quiescent ECs, previous studies indicate that these integrins are tumor vasculature markers [46]. This evidence suggests that the vessel-like structures observed in the lung metastases are, indeed, blood vessels expressing β_3_ and β_5_ integrins. Double immunostaining for β_3_ and β_5_ integrins with endomucin revealed their co-localization in these vessel-like structures, confirming that both integrins are expressed in the tumor vasculature (Supplementary Fig. 1A).

Hence, our results demonstrate that 4T1 and RENCA lung metastases can be targeted by cilengitide, as an inhibitor of α_v_β_3_, α_v_β_5_ and α_5_β_1_ integrins [42]. Interestingly, the main ligands for these integrins, fibronectin (ligand for β_1_, β_3_, and β_5_ integrins) and collagen I (ligand for β_1_ integrin), have higher expression in 4T1 compared to RENCA lung metastases (Supplementary Fig. 1B and C).

### Cilengitide enhances tumor progression in lung metastases characterized by angiogenesis-driven vascularization

**Fig. 3:**
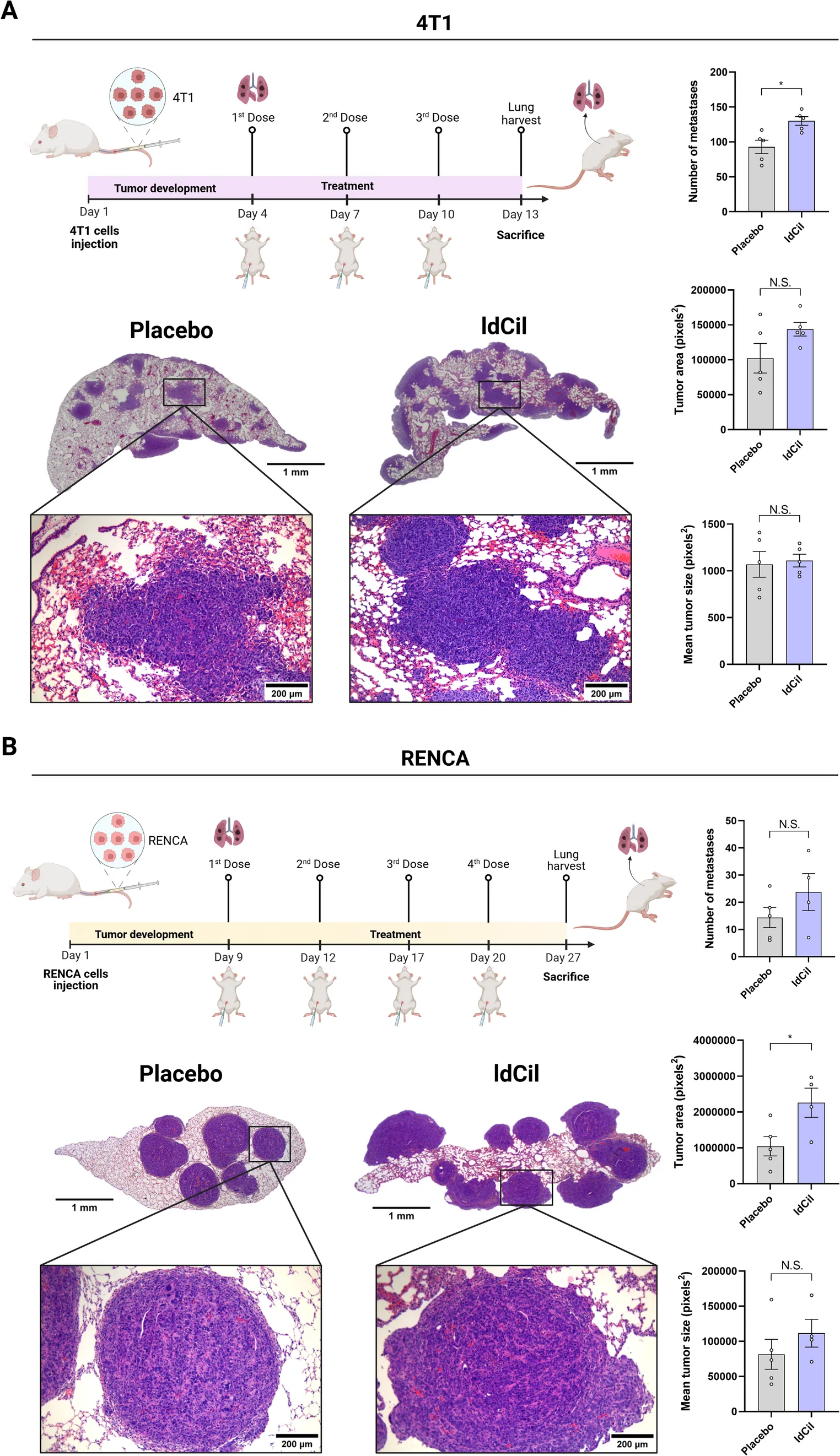
Treatment schedule and effect of cilengitide on 4T1 and RENCA lung metastases growth. **A** *VCO lung metastases*. The left panel shows the treatment calendar in 4T1 VCO lung metastases in BALB/c mice and representative pictures of lung lobes bearing 4T1 metastases treated with PBS or 50 µg/kg cilengitide stained with H&E. The background of the H&E staining images has been removed using PowerPoint in order to clarify the observation of the lung metastases. The right panel shows data analysis of the number of tumors, tumor area and mean tumor size of placebo and ldCil-treated 4T1 lung metastases (*n* = 5). Individual values are represented in the graph and the bars represent the mean ± SEM. Scale bar = 1 mm (gross lung) and 200 μm (zoomed area). **B** *Angiogenic lung metastases*. The left panel shows the treatment schedule in RENCA angiogenic lung metastases in BALB/c mice and representative pictures of lung lobes bearing RENCA metastases treated with PBS or 50 µg/kg cilengitide stained with H&E. The background of the H&E staining images has been removed using PowerPoint in order to clarify the observation of the lung metastases. The right panel shows data analysis of the number of tumors, tumor area and mean tumor size of placebo and ldCil-treated RENCA lung metastases (*n* = 5 in placebo and *n* = 4 in ldCil). Individual values are represented in the graph and the bars represent the mean ± SEM. Scale bar = 1 mm (gross lung) and 200 μm (zoomed area). Schemes created with BioRender.com (license: DS2A37KMR2). N.S., non-statistical differences; * *p* ≤ 0.05. Student’s *t* test (**A** and **B**)

The analysis of mouse lung bearing 4T1 VCO lung metastases demonstrated that ldCil treatment did not change either mean tumor size or tumor area, although it increased the number of lesions (Fig. 3A). However, in RENCA lung metastases, in which tumor growth is driven by angiogenesis, we observed an increase in tumor area, but no significant changes in either the number of lesions or the mean tumor size (Fig. 3B). These data suggest that ldCil induces different effects in lung metastases depending on the HGP.

Afterwards, we sought to investigate a possible distinct direct effect of ldCil on tumor cells biology that could account for the differences observed in tumor growth in vivo between 4T1 and RENCA lung metastases. First, we observed a similar reduction of cell viability of 4T1 and RENCA after cilengitide treatment at different low concentrations (Supplementary Fig. 2A). In addition, Ki67 immunostaining did not reveal any difference in 4T1 and RENCA cell proliferation after ldCil treatment (Supplementary Fig. 2B). These data demonstrate that cilengitide does not stimulate tumor cell proliferation at any concentration. Therefore, this confirms that the enhanced tumor growth observed under ldCil treatment in vivo must be driven by the remodeling of the TME or the tumor vasculature, rather than any direct proliferative effect on the cancer cells. On the other hand, the migration assay results in 4T1 and RENCA cell lines demonstrated that cilengitide does not increase cell migration (Supplementary Fig. 2C). However, analyzing cell adhesion to extracellular matrix (ECM) components, we found that ldCil reduces 4T1 cell adhesion to collagen I and fibronectin, and to fibronectin in RENCA cells (Supplementary Fig. 2B). Since β_3_ and β_5_ integrins are almost absent in cancer cells as described previously, the effect of cilengitide on tumor cells may be regulated by β_1_ integrin, that also shows specificity for cilengitide at a IC_50_ of 14.9 nM [42]. We observed that fibronectin (an RGD domain-containing ligand for β_1_, β_3_, and β_5_ integrins) and collagen I (also a ligand for β_1_ integrin) are highly abundant in 4T1 metastatic lesions, where the increase in the number of metastases upon ldCil treatment is prominent (Supplementary Fig. 1B). Conversely, their expression is substantially lower in the RENCA model, where no significant increase in number of metastases was detected.

Additionally, we functionally demonstrated that 4T1 and RENCA cells adhesion to fibronectin is α_5_β_1_ integrin dependent. In 4T1 cell line, adhesion to fibronectin is only blocked by a specific inhibitor of this integrin, c(phg-isoDGR-(NMe)k) TFA (isoDGR) [47], but there is not an additional inhibitory effect when combining isoDGR with ldCil (Supplementary Fig. 2D). In the case of RENCA cell line, the inhibition of adhesion induced by cilengitide treatment is lost upon inhibition of β_1_ integrin with isoDGR (Supplementary Fig. 2D). On the other hand, isoDGR did not change 4T1 cells adhesion to collagen I, suggesting that this process is α_5_β_1_ integrin independent.

Given the preferential affinity of cilengitide for α_v_β_3_ (IC_50_ = 0.61 ± 0.06 nM) and α_v_β_5_ integrins (IC_50_ = 8.4 ± 2.1 nM) and the predominant expression of these receptors in ECs, the effects of ldCil are expected to be primarily mediated through the tumor vasculature [42]. As its affinity for α_5_β_1_ is substantially lower (IC_50_ = 14.9 ± 3.1 nM), it suggests that any direct effect on β_1_ integrin signalling is likely to be modest. Nonetheless, an effect of ldCil on tumor cell-ECM interactions cannot be excluded. In ECM rich tumors, such as 4T1 metastases, previous studies have shown that β_1_ integrin deletion promotes tumor cell detachment from the basement membrane in breast cancer models [48]. As a consequence, cell adhesion to ECM may be reduced, facilitating cell detachment and dissemination, without necessarily affecting the temporal kinetics of metastatic seeding.

### ldCil differentially modulates vascular density, pericyte coverage and hypoxia in 4T1 and RENCA lung metastases

**Fig. 4:**
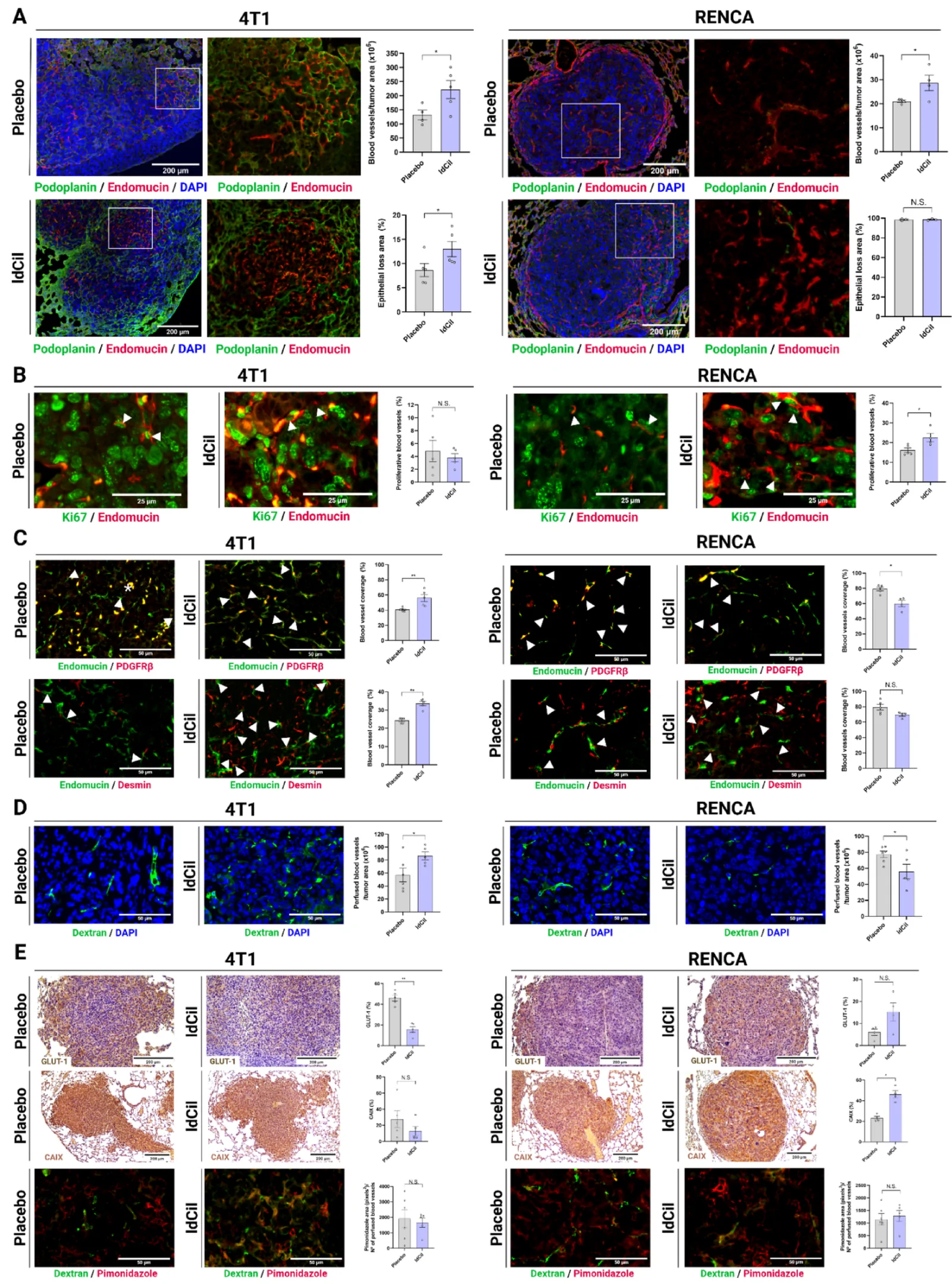
Effect of cilengitide on tumor blood vessels and hypoxia in 4T1 and RENCA lung metastases. **A** *Endomucin/podoplanin staining in 4T1 and RENCA lung metastases.* The left panel shows representative pictures of 4T1 metastases treated with PBS or 50 µg/kg cilengitide stained for endomucin (red), podoplanin (green) and cell nuclei (blue) detection. The squares represent the areas that have been magnified. Blood vessel density and *Epithelial Loss Area* analysis are also included (*n* = 5). Blood vessel density has been represented by multiplying the blood vessel/tumor area ratio by the correction factor 10^6^. Individual values are represented in the graph and the bars represent the mean ± SEM. The right panel shows representative pictures of RENCA lung metastases treated with PBS or 50 µg/kg cilengitide stained for endomucin (red), podoplanin (green) and cell nuclei (blue) detection. Blood vessel density analysis and *Epithelial Loss Area* are also included (*n* = 5 in placebo and *n* = 4 in ldCil). Blood vessel density has been represented by multiplying the blood vessel/tumor area ratio by the correction factor 10^6^. Individual values are represented in the graph and the bars represent the mean ± SEM. Scale bar = 200 μm. **B** *Ki67 and endomucin double immunofluorescence.* The left panel shows representative pictures of 4T1 metastases treated with PBS or 50 µg/kg cilengitide stained for Ki67 (green) and endomucin (red). Double Ki67^+^ and endomucin^+^ cells are considered proliferating ECs. Analysis of the percentage of proliferating ECs is included (*n* = 5). Individual values are represented in the graph and the bars represent the mean ± SEM. The right panel shows representative pictures of RENCA metastases treated with PBS or 50 µg/kg cilengitide stained for Ki67 (green) and endomucin (red). Double Ki67^+^ and endomucin^+^ cells are considered proliferating ECs. Analysis of the percentage of proliferating ECs is included (*n* = 5 in placebo and *n* = 4 in ldCil). Individual values are represented in the graph and the bars represent the mean ± SEM. White arrows point out proliferative blood vessels. Scale bar = 25 μm. **C** *Endomucin/PDGFRβ staining and endomucin/desmin staining in 4T1 and RENCA lung metastases.* The left panel shows representative pictures of 4T1 metastases treated with PBS or 50 µg/kg cilengitide stained for endomucin (green) and PDGFRβ (red) detection or endomucin (green) and desmin (red) detection. White arrows point out blood vessels covered with pericytes. Yellow signal (*) corresponds to the presence of erythrocytes. The analysis of pericyte coverage using both markers is also included (*n* = 5). Individual values are represented in the graph and the bars represent the mean ± SEM. The right panel shows representative pictures of RENCA metastases treated with PBS or 50 µg/kg cilengitide stained for endomucin (green) and PDGFRβ (red) detection and endomucin (green) and desmin (red) detection. White arrows point out blood vessels covered with pericytes. The analysis of pericyte coverage is also included (*n* = 5 in placebo and *n* = 4 in ldCil). Individual values are represented in the graph and the bars represent the mean ± SEM. Scale bar = 50 μm. **D** *Blood vessel perfusion*. The left panel shows representative pictures of FITC-dextran perfused vessels (green) in 4T1 metastases treated with PBS or 50 µg/kg cilengitide. The analysis of the number of perfused blood vessels per tumor area is included (*n* = 6). The number of perfused blood vessels per tumor area has been represented by multiplying by the correction factor 10^6^. Individual values are represented in the graph and the bars represent the mean ± SEM. The right panel shows representative pictures of FITC-dextran perfused vessels (green) in RENCA metastases treated with PBS or 50 µg/kg cilengitide. The analysis of the number of perfused blood vessels per tumor area is included (*n* = 6). Individual values are represented in the graph and the bars represent the mean ± SEM. Scale bar = 50 μm. **E** *Hypoxia levels determined by GLUT-1*,* CAIX and pimonidazole detection in 4T1 and RENCA lung metastases.* The left panel shows representative pictures of 4T1 metastases treated with PBS or 50 µg/kg cilengitide stained for GLUT-1, CAIX and pimonidazole detection. Data analysis of GLUT-1 (*n* = 5), CAIX (*n* = 5) and pimonidazole (*n* = 6 in placebo and *n* = 5 in ldCil) percentages are also included. Individual values are represented in the graph and the bars represent the mean ± SEM. The right panel shows representative pictures of RENCA metastases treated with PBS or 50 µg/kg cilengitide stained for GLUT-1, CAIX and pimonidazole detection. Data analysis of GLUT-1 (*n* = 4), CAIX (*n* = 4) and pimonidazole (*n* = 6 in placebo and *n* = 5 in ldCil) percentages are also included. Individual values are represented in the graph and the bars represent the mean ± SEM. Scale bar GLUT-1 and CAIX stainings = 200 μm. Scale bar pimonidazole staining = 50 μm. N.S., non-statistical differences; * *p* ≤ 0.05; ** *p* ≤ 0.01. Student’s *t* test for blood vessel density, perfused blood vessels and pimonidazole/number of perfused blood vessels analysis and Mann-Whitney *U* test for the analysis of *Epithelial Loss Area*, proliferative blood vessels percentage, pericyte coverage, GLUT-1 and CAIX percentage

To understand possible vascular changes induced by ldCil, endomucin immunofluorescence analysis revealed a statistically significant increment in the number of blood vessels per tumor area in both experimental models after ldCil treatment (Fig. 4A). In the 4T1 model, we also observe an increase in *Epithelial Loss Area* after ldCil, whereas no changes are seen in the RENCA model (Fig. 4A). The initial number of blood vessels is higher in the 4T1 model due to the different HGP of each model in basal conditions: whereas in 4T1 metastases pre-existing blood vessels are preserved, in the RENCA ones, tumor cells push lung structure and pre-existing vessels are compressed and displaced to the border of the tumor [5, 10]. To confirm the non-proliferative or proliferative state of ECs, we performed double immunofluorescence of Ki67 and endomucin, and we quantified the percentage of endomucin positive and Ki67 cells. The analysis shows that this number is very low in VCO tumors (around 4%) confirming the quiescent state of co-opted blood vessels. More interestingly we did not find any statistical significance in 4T1 lung metastases treated with ldCil (Fig. 4B). This result is important because it indicates that the increased blood vessel density is not due to enhanced vessel branching, but rather to the survival of co-opted blood vessels after a period in which they undergo regression [49]. However, in RENCA lung metastases we observe a higher percentage of proliferating ECs (more than 15%), confirming the proliferative phenotype of angiogenic vessels, being this number higher in RENCA lung metastases treated with ldCil (Fig. 4B).

In respect of the blood vessel structure, we analyzed pericyte coverage to determine their maturity using two different pericyte markers: PDGFRβ and desmin [33]. We found that ldCil promotes a significant increase in pericyte coverage in 4T1 metastases and a significant decrease in RENCA lung metastases after ldCil treatment (Fig. 4C). By analyzing FITC-dextran perfusion, we observed an increase in the number of functional blood vessels after ldCil treatment in the 4T1 model. However, we observed a decrease in the number of functional blood vessels after ldCil in the RENCA model (Fig. 4D).

The normalized vasculature observed in 4T1 lung metastases after ldCil treatment is associated with a reduction in tumor hypoxia, as determined by the significant decrease in the GLUT-1 marker. A similar, although non-significant, trend was observed for CAIX, whereas no differences were detected for pimonidazole (Fig. 4E). On the other hand, the immature blood vessels formed after ldCil in RENCA lung metastases are associated with higher levels of hypoxia, evidenced by the significant increase in the CAIX marker, which was accompanied by a non-significant upward trend in GLUT-1 marker. As observed in the 4T1 model, no differences in the pimonidazole area after ldCil treatment were detected (Fig. 4E). The literature has long established that GLUT-1 and CAIX are markers of chronic or diffusion-limited hypoxia [50, 51], whereas pimonidazole is closely associated with acute or transient hypoxic events [52]. These findings imply that the microenvironmental differences between 4T1 and RENCA metastases are driven by their long-term chronic hypoxic history rather than immediate, real-time perfusion disparities.

Taken together, these data indicate that ldCil differentially modulates co-opted versus angiogenic blood vessels. In VCO lung metastases, ldCil promotes vessel normalization, as evidenced by an increase in pericyte coverage without an elevation in ECs proliferation, alongside improved vessel perfusion and attenuated tumor hypoxia. Conversely, in angiogenic lung metastases, ldCil induces non-functional angiogenesis. This is characterized by increased blood vessel density and ECs proliferation, coupled with a downward trend in pericyte coverage and vessel perfusion, ultimately leading to exacerbated tumor hypoxia.

We also checked the effect of ldCil on highly vascularized tissues such as kidneys in free-tumor healthy mice. Treatment with ldCil does not modify the renal ultrastructure, as we do not observe changes in glomerular morphology, hypercellularity and tubule atrophy, evaluated by H&E staining (Supplementary Fig. 3A). Moreover, looking at kidneys from ldCil treated animals we do not observe changes in capillary area in the glomeruli and the renal tubular interstitium (Supplementary Fig. 3B).

### ldCil treatment enhances ECs barrier function in a VEGF-independent manner and induces VEGF-driven angiogenesis

ldCil has been shown to promote tumor angiogenesis through a mechanism involving upregulation of VEGFR2 levels, thereby rendering its pro-migratory effects on ECs largely VEGF-dependent [26]. In contrast, VCO is characterized by a quiescent endothelial phenotype associated with low VEGFR2 expression [10]. Given that co-opted vessels are generally refractory to VEGF signaling and anti-VEGF therapies [5, 8–10, 19], we sought to evaluate whether ldCil could modulate endothelial stability in the absence of VEGF.

**Fig. 5:**
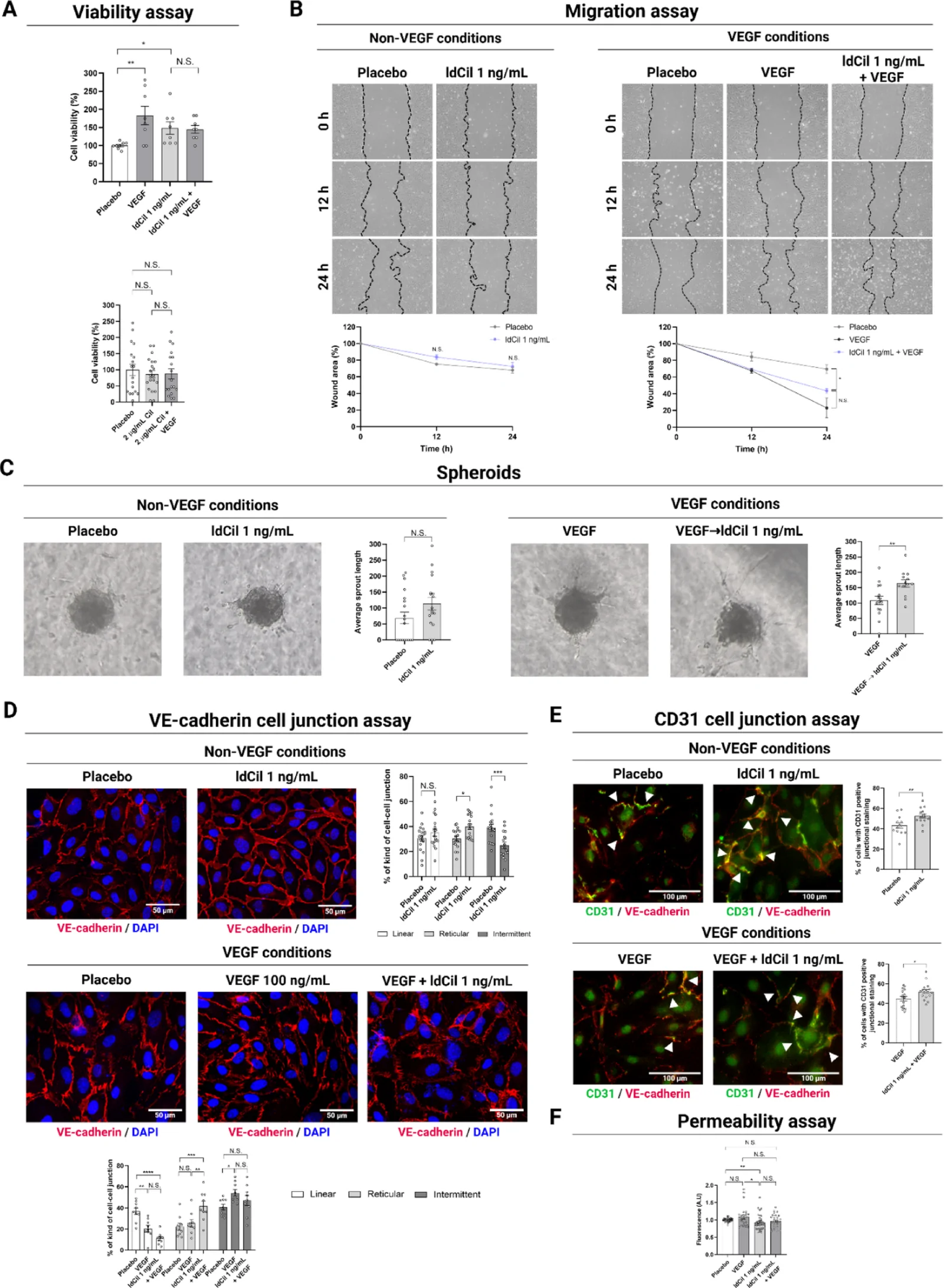
Effect of cilengitide in HUVECs viability,*migration*, sprouting, cell-cell junctions and vascular permeability. **A** *Cell viability percentage of HUVECs cell line treated with different concentrations of cilengitide in the presence or absence of 100 ng/mL VEGF.* Upper panel displays HUVECs viability when treated with a low dose of cilengitide (1 ng/mL), whereas lower panel shows cell viability in presence of a high dose of cilengitide (2 µg/mL). Individual values are represented in the graph and the bars represent the mean ± SEM. **B** *Migration capacity of HUVECs cells treated with 1 ng/mL cilengitide in presence or absence of 100 ng/mL VEGF.* Representative pictures of the wound healing assays performed in HUVECs treated with cilengitide in VEGF-free conditions or VEGF conditions are shown. Data analysis of the wound area percentage is included. In the graph, each point represents the mean ± SEM. All groups were compared at every time point. **C** *Endothelial sprouting capacity of HUVECs spheroids treated with 100 ng/mL VEGF in monotherapy or in combination with 1 ng/mL cilengitide.* Representative pictures of the spheroids assay are shown. Sprout length analysis is also included. Individual values are represented in the graph and the bars represent the mean ± SEM. **D** *Percentage of linear*,* reticular and intermittent cell-cell junctions in HUVECs treated with 1 ng/mL cilengitide in presence or absence of 100 ng/mL VEGF.* Representative pictures of HUVECs cell line stained for VE-cadherin (red) detection in non-VEGF conditions or VEGF-conditions are displayed. Data analysis of the kind of cell junctions percentage is also included. Individual values are represented and the bars represent the mean ± SEM. Scale bar = 50 μm. **E** *CD31 and VE-Cadherin double immunofluorescence in HUVECs treated with 1 ng/mL cilengitide in presence or absence of 100 ng/mL VEGF*. Representative pictures of HUVECs cell line stained for CD31 (green) and VE-cadherin (red) detection in non-VEGF conditions or VEGF-conditions are displayed. Data analysis of CD31 cell junctions percentage is also included. Individual values are represented and the bars represent the mean ± SEM. White arrows point out CD31 positive junctions that are considered reticular. Scale bar = 100 μm. **F** *Permeability assay of HUVECs cells monolayer treated with 1 ng/mL cilengitide using FITC-dextran in combination or not with 100 ng/mL VEGF.* Panel represents data analysis of FITC-dextran fluorescence along HUVECs monolayer at 6 h. Fluorescence intensity is directly proportional to cell permeability. Individual values are represented in the graph and the bars represent the mean ± SEM. N.S., non-statistical differences; * *p* ≤ 0.05; ** *p* ≤ 0.01; *** *p* ≤ 0.001; **** *p* ≤ 0.0001. Kruskal Wallis test, post hoc Dunn’s test (**A**, **F**); Two-way ANOVA, post hoc Tukey’s test (**B**, **D**); Student’s *t* test (**C**); Mann-Whitney *U *test (**E**)

Cell viability assays performed in HUVECs revealed that, under VEGF-deprived conditions, cilengitide modestly increases cell viability at 1 ng/mL, while it does not alter VEGF-induced viability (Fig. 5A). Moreover, we performed another viability assay using a high dose of cilengitide (2 µg/mL) and no differences were observed (Fig. 5A), demonstrating that cilengitide only exerts an effect on improving ECs survival at low doses. On the other hand, wound healing assays performed with HUVECs in presence and in absence of VEGF demonstrated that ECs migration is not increased by cilengitide alone but in combination with VEGF (Fig. 5B). To assess the cooperative effects of cilengitide and VEGF on angiogenic behavior, we performed a sprouting assay in HUVECs spheroids. In this setting, cilengitide at 1 ng/mL significantly potentiated VEGF-induced sprout elongation, indicating a synergistic pro-angiogenic interaction (Fig. 5C).

Beyond its effects on endothelial activation, we investigated the impact of ldCil on ECs VE-cadherin junctions organization. In the absence of VEGF, cilengitide at 1 ng/mL does not significantly alter the abundance of linear junctions but induces a marked increase in reticular junctions concomitant with a reduction in intermittent junctions (Fig. 5D). This redistribution is indicative of enhanced junctional maturation and endothelial stabilization under VEGF-independent conditions [53–55]. As expected, VEGF treatment reduced linear junctions. However, this effect was not further modulated by cilengitide. Notably, reticular junctions were significantly increased upon combined VEGF and ldCil treatment, whereas intermittent junctions remained unchanged [56] (Fig. 5D). To confirm the increase in ECs reticular junctions after ldCil treatment we analysed CD31 in HUVECs cell-cell junctions. The presence of CD31 in the ECs junctions has been associated with a reticular phenotype [57]. We show an effect of cilengitide 1 ng/ml in enhancing the percentage of cells with CD31^+^ junctional staining in absence and in presence of VEGF (Fig. 5E). Collectively, these data indicate that ldCil promotes VE-cadherin junctional stabilization both in absence and in presence of VEGF, primarily by shifting the balance towards reticular junctional architectures. Consistent with this, endothelial permeability was reduced, as demonstrated by decreased FITC-dextran leakage in HUVECs monolayers treated with cilengitide at 1 ng/mL (Fig. 5F). However, there is no difference in HUVECs permeability upon VEGF alone in comparison with the combination of VEGF and ldCil, indicating that the reduced permeability occurs only in absence of VEGF (Fig. 5F).

Taken together, these data indicate that ldCil promotes endothelial junctional stabilization, providing a mechanistic basis for the vascular changes observed in 4T1 VCO tumors. In these lesions, vascularization predominantly occurs independently of VEGF/VEGFR2 signaling [58], is associated with low VEGF levels and involves blood vessels exhibiting a quiescent endothelial phenotype [10]. In this context, ldCil treatment favors the formation of a fine and organized vascular network, consistent with the increased endothelial cell viability observed under VEGF-deprived conditions, the elevated vessel density observed in the in vivo model and the improved endothelial barrier function. Importantly, although VCO happens under vascularization mechanisms independent of VEGF/VEGFR2 axis, the effect of ldCil on enhancing vessel normalization in 4T1 lung metastases may also depend, at some extent, on VEGF/VEGFR2 signaling, as demonstrated by its abrogation upon administration of the anti-angiogenic agent sorafenib [59] (Supplementary Fig. 4A and B). VEGF has been shown to play a critical role in blood vessel viability. This VEGF is likely expressed by pericytes, which support and stabilize the vasculature while promoting endothelial cell survival via the induction of autocrine VEGF-A signaling [60, 61].

In the RENCA model, no differences in the number of metastases or tumor area were observed between sorafenib alone and combined with ldCil, but mean tumor size was increased significantly with the combination of sorafenib and ldCil (Supplementary Fig. 4C). In addition, the perimeter-to-area ratio was significantly reduced with the combined treatment, indicating a shift towards a more rounded morphology, characteristic of the pushing HGP. Consistent with this finding, sorafenib-treated metastases displayed a predominantly co-optive phenotype, with 73% exhibiting an alveolar-interstitial HGP compared with 15% in the placebo group (Fig. 1B). Taken together, these results indicate that ldCil treatment reverses the co-optive phenotype induced by sorafenib, by increasing the percentage of metastases with pushing HGP. (Supplementary Fig. 4D). On the other hand, we did not observe differences in blood vessel density due to the effect of sorafenib in inhibiting VEGF/VEGFR2 and thus blocking the vascular effects of ldCil. (Supplementary Fig. 4D).

Our findings indicate that ldCil can potentiate angiogenic expansion only when VEGF signaling is functionally available. In fact, in angiogenic RENCA metastases, ldCil treatment induces features characteristic of non-productive tumor angiogenesis, including an increased vessel density associated with reduced pericyte coverage and exacerbated tumor hypoxia. These observations underscore the context-dependent effects of ldCil on tumor vasculature, promoting vessel stabilization and functional remodeling in VCO tumors while exacerbating vascular dysfunction in VEGF-driven angiogenic settings.

### ldCil treatment has limited effects on the TIME in both non-angiogenic and angiogenic lung metastases

**Fig. 6:**
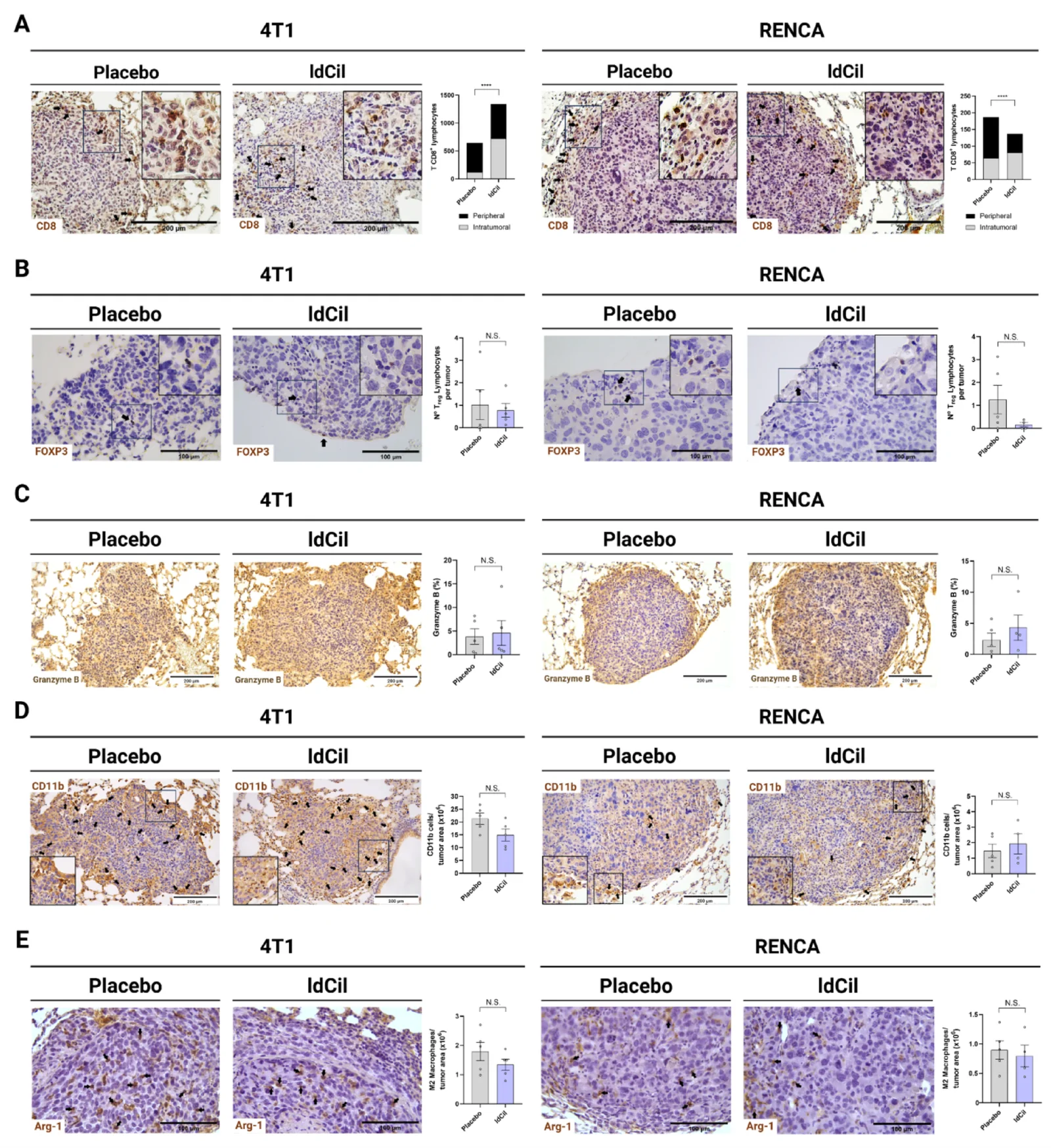
Tumor immune microenvironment (TIME) changes induced by cilengitide treatment in 4T1 and RENCA lung metastases. **A** *T CD8*^+^ *lymphocytes infiltration and distribution in 4T1 and RENCA lung metastases*. The left panel shows representative pictures of 4T1 tumors treated with PBS or 50 µg/kg cilengitide stained for CD8 receptor detection. Data analysis of the number of T CD8^+^ cells is also included. The right panel shows representative images of RENCA tumors treated with PBS or 50 µg/kg cilengitide stained for CD8 receptor detection. Data analysis of the number of T CD8^+^ cells is also included. Black arrows point out T CD8^+^ lymphocytes and the squares represent the areas that have been magnified. Scale bar = 200 μm. **B** *Abundance of regulatory T lymphocytes in 4T1 and RENCA lung metastases*. The left panel shows representative pictures of 4T1 tumors treated with PBS or 50 µg/kg cilengitide stained for FOXP3 marker. Data analysis of the number of T regulatory cells per tumor is also included (*n* = 5). The right panel shows representative images of RENCA tumors treated with PBS or 50 µg/kg cilengitide stained for FOXP3 detection. Data analysis of the number of T regulatory cells per tumor is also included (*n* = 5 placebo and *n* = 4 ldCil). Individual values are represented in the graph and the bars represent the mean ± SEM. Black arrows point out T_reg_ lymphocytes and the squares represent the areas that have been magnified. Scale bar = 100 μm. **C** *Granzyme B immunohistochemical localization in 4T1 and RENCA lung metastases*. The left panel shows representative pictures of 4T1 tumors treated with PBS or 50 µg/kg cilengitide stained for granzyme B detection. Data analysis of the percentage of granzyme B is also included (*n* = 5). The right panel shows representative images of RENCA tumors treated with PBS or 50 µg/kg cilengitide stained for granzyme B detection. Data analysis of the percentage of granzyme B is also included (*n* = 5 placebo and *n* = 4 ldCil). Scale bar = 200 μm. **D** *Abundance of CD11b*^+^ *myeloid cells in 4T1 and RENCA lung metastases.* The left panel shows representative pictures of 4T1 tumors treated with PBS or 50 µg/kg cilengitide stained for CD11b detection. Data analysis of the number of CD11b^+^ myeloid cells per tumor area is also included (*n* = 5). The number of CD11b^+^ myeloid cells per tumor area has been represented by multiplying by the correction factor 10^6^. Individual values are represented in the graph and the bars represent the mean ± SEM. The right panel shows representative images of RENCA tumors treated with PBS or 50 µg/kg cilengitide stained for CD11b detection. Data analysis of the number of CD11b^+^ myeloid cells per tumor is also included (*n* = 5 placebo and *n* = 4 ldCil). The number of CD11b^+^ myeloid cells per tumor area has been represented by multiplying by the correction factor 10^6^. Individual values are represented in the graph and the bars represent the mean ± SEM. Black arrows point out CD11b^+^ cells and the squares represent the areas that have been amplified. Scale bar = 200 μm. **E** *M2 macrophages infiltration in 4T1 and RENCA lung metastases*. The left panel shows representative pictures of 4T1 tumors treated with PBS or 50 µg/kg cilengitide stained for Arginase-1 (M2 macrophage marker) detection. Black arrows point out M2 macrophages. The analysis of M2 macrophage infiltration is also included in the figure (*n* = 5). The number of M2 macrophages per tumor area has been represented by multiplying by the correction factor 10^6^. Individual values are represented in the graph and the bars represent the mean ± SEM. The right panel shows representative images of RENCA tumors treated with PBS or 50 µg/kg cilengitide stained with immunohistochemistry for Arginase-1 detection. The analysis of tumor M2 macrophages is also included in the figure (*n* = 5 placebo and *n* = 4 ldCil). The number of M2 macrophages per tumor area has been represented by multiplying by the correction factor 10^6^. Individual values are represented in the graph and the bars represent the mean ± SEM. Black arrows point out M2 macrophages. Scale bar = 100 μm. N.S., non-statistical differences; **** *p* ≤ 0.0001. Fisher’s exact test (**A**); Student’s *t* test (**B**, **D**, **E**); Mann-Whitney *U *test (**C**)

To evaluate the impact of ldCil treatment on the TIME, we analyzed several immune components within lung metastases by immunostaining approaches, allowing us to assess both the abundance and the spatial distribution of immune cells. First, we demonstrated that ldCil increases CD8^+^ T lymphocyte infiltration in 4T1 VCO lung metastases (Fig. 6A), suggesting that the fine vascular network of blood vessels allows lymphocytes penetration into the lesions core. However, in RENCA lung metastases, the total number of CD8^+^ T lymphocytes decreased. Notably, ldCil increased intratumoral lymphocyte infiltration, although to a lesser extent than that observed in the VCO model (Fig. 6A).

On the other hand, ldCil administration did not alter the number of regulatory T cells (T_reg_) in either model (Fig. 6B). Consistent with these outcomes, granzyme B levels remained unchanged following ldCil treatment, indicating that changes in CD8^+^ T-cell infiltration were not accompanied by detectable changes in cytotoxic effector activity at the analyzed time point in both 4T1 and RENCA metastases (Fig. 6C).

With respect to the myeloid cells, identified by the marker CD11b, we did not observe differences after ldCil treatment either in 4T1 or RENCA lung metastases (Fig. 6D). Nevertheless, myeloid cells displayed distinct spatial distributions in the two models, being predominantly located within the tumor core of 4T1 metastases and mainly confined to the tumor periphery in RENCA metastases (Fig. 6D). Finally, no significant differences were detected in the number of M2 macrophages either in 4T1 or RENCA metastases after ldCil administration (Fig. 6E).

Collectively, these findings indicate that, despite the extensive vascular normalization triggered by ldCil in VCO metastases, the subsequent alterations within the TIME remained notably modest, with the exception of enhanced CD8^+^ T-cell infiltration. Particularly, in RENCA angiogenic tumors, characterized by defective and aberrant angiogenesis after ldCil treatment, the absence of significant immune changes is consistent with the persistent vascular dysfunction and limited capacity to support efficient immune cell infiltration.

### The normalized vasculature in non-angiogenic lung metastases enhances chemotherapy efficacy

In order to evaluate the real effectiveness of ldCil treatment in our two experimental lung metastases models, BALB/c mice were treated with a combination of ldCil and the chemotherapy agent Ptax^5^ in both 4T1 and RENCA metastases as well as Carbo^5^ in 4T1 lung metastases.

**Fig. 7:**
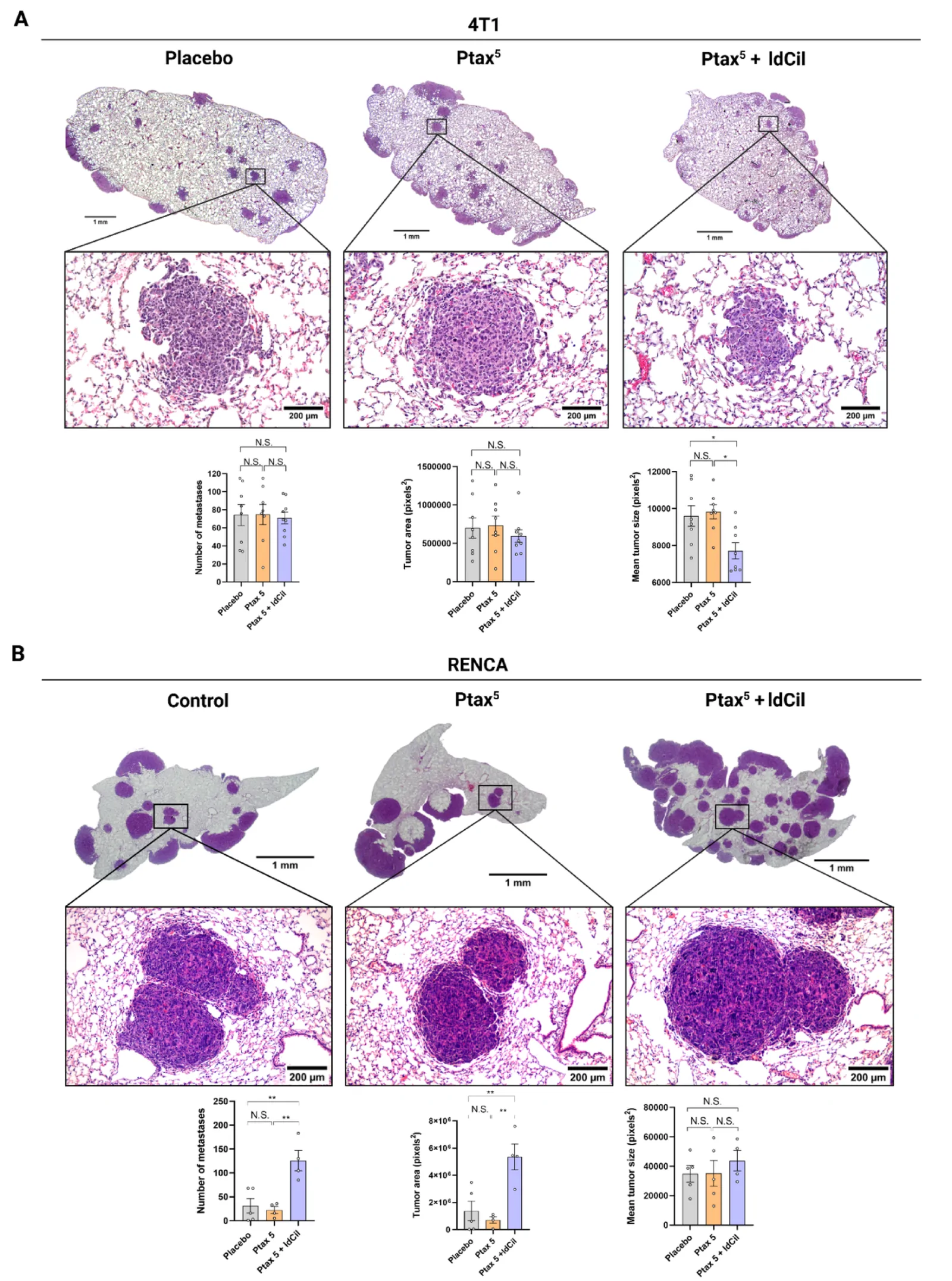
Effect of cilengitide on paclitaxel efficacy in 4T1 and RENCA lung metastases. **A** *Number of metastases*,* tumor area and mean tumor size after corn-oil (placebo)*,* 5 mg/kg paclitaxel (Ptax*^5^*) and ldCil combined with Ptax*^5^ *treatments in 4T1 experimental lung metastases*. The upper panel shows representative images of a mouse lung lobe bearing 4T1 metastasis treated with corn-oil (placebo), Ptax^5^ or Ptax^5^ combined with 50 µg/kg cilengitide stained with H&E. The background of the H&E staining images has been removed using PowerPoint in order to clarify the observation of the lung metastases. The lower panel shows data analysis of the number of metastases, tumor area and mean tumor size. Data analysis of the number of tumors, tumor area and mean tumor size of placebo, Ptax^5^ or Ptax^5^ with ldCil lung metastases is also included (*n* = 8 placebo and Ptax^5^; *n* = 9 Ptax^5^ in combination with ldCil). Individual values are represented in the graph and the bars represent the mean ± SEM. Scale bar = 1 mm (gross lung) and 200 μm (zoomed area). **B** *Number of metastases*,* tumor area and mean tumor size after corn-oil (placebo)*,* 5 mg/kg paclitaxel (Ptax*^5^*) and ldCil combined with Ptax*^5^ *treatments in RENCA experimental lung metastases*. The upper panel shows H&E-stained images of a mouse lung lobe with RENCA metastases treated with corn-oil, Ptax^5^ or Ptax^5^ combined with 50 µg/kg cilengitide. The background of the H&E staining images has been removed using PowerPoint in order to clarify the observation of the lung metastases. The lower panel shows data analysis of the number of metastases, tumor area and mean tumor size. Data analysis of the number of tumors, tumor area and mean tumor size of placebo, Ptax^5^ or Ptax^5^ with ldCil lung metastases is also included (*n* = 5 placebo; Ptax^5^ and Ptax^5^ with ldCil *n* = 4). Scale bar = 1 mm (gross lung) and 200 μm (zoomed area). N.S., Non-statistical differences; * *p* ≤ 0.05; ** *p* ≤ 0.01. One-way ANOVA test, post hoc test Sidak (**A** and **B**)

Our results indicate that the combination treatment does not significantly reduce either the number of metastases or the tumor area compared to placebo and paclitaxel monotherapy in VCO tumors (Fig. 7A). Even so, the combined treatment with ldCil and Ptax^5^ resulted in a significant reduction in mean tumor size compared with both placebo and paclitaxel monotherapy (Fig. 7A). The in vitro viability assay performed in 4T1 cells combining different doses of paclitaxel with cilengitide 1 ng/mL did not show any changes with respect to ldCil treatment alone (Supplementary Fig. 5A) and thus, the reduction of mean tumor size observed in the mouse model is due to changes in the vascular network and not to an increase in chemosensitization of ldCil.

In contrast, the dual combination of ldCil and paclitaxel in the RENCA model significantly increased the number of metastases and tumor area in compared with placebo and paclitaxel monotherapy (Fig. 7B). We compared these results with a viability assay performed in RENCA cells treated with different doses of paclitaxel in presence or absence of 1 ng/mL cilengitide. Overall, the presence of cilengitide reduced the cytotoxic effect of paclitaxel at most of the doses tested (Supplementary Fig. 5A).

The effects observed with paclitaxel in the 4T1 model were also reproduced when using Carbo^5^ in combination with ldCil. We detected a significant reduction in mean tumor size compared with Carbo^5^ as monotherapy (Supplementary Fig. 5B), demonstrating that ldCil also enhances the antitumor efficacy of an alternative chemotherapy regimen in the 4T1 model. This result was compared with a viability assay carried out in the 4T1 cell line treated with different doses of carboplatin in combination with 1 ng/mL cilengitide. Differences between the combined treatment and carboplatin monotherapy emerged only at higher carboplatin concentrations, where the combination was associated with significantly higher cell viability (Supplementary Fig. 5C).

## Discussion

In this study, we demonstrate that ldCil differentially remodels tumor vasculature in two different experimental models of lung metastases with different HGP. Whereas ldCil normalizes the vascular network in VCO metastases, it induces an expanded yet immature and dysfunctional vascular network in metastases that initially exhibit angiogenic growth. By treating non-angiogenic lung metastases with ldCil we observed the restoration of the co-opted vasculature after the regression triggered by its VCO tumor growth, as evidenced by the increased number of functional blood vessels, improved ECs junctions and higher pericyte coverage, creating a fine vascular network. Thus, the combination of ldCil and chemotherapy shows the ability to reduce mean tumor size. In contrast, in RENCA lung metastases, which undergo angiogenesis, ldCil increases tumor angiogenesis leading to a defective blood vessel network associated with the lack of chemotherapy response.

VCO tumors are intrinsically insensitive to AAT, and tumors that initially rely on angiogenesis can switch to VCO growth after AAT, thereby acquiring treatment resistance [8, 12]. However, until now, no agent to inhibit VCO growth or to modulate co-opted blood vessels has been described. Single cell analyses have shown that ECs and pericytes from VCO metastases generated by the injection of 4T1 cells exhibited a quiescent phenotype, similar to non-tumoral blood vessels, suggesting limited responsiveness to anti-VEGF strategies [10]. This was validated by Bridgeman et al. who demonstrated that VCO lung metastases were not responsive to the VEGFR-tyrosine kinase inhibitor sunitinib [5]. However, despite this quiescent phenotype, co-opted vessels are not functionally normal, as they undergo progressive compression and regression by tumor expansion [21, 49].

Based on these observations, we hypothesized that, rather than suppressing vascularization, restoring the integrity and functional competence of the pre-existing vascular network could represent a more effective strategy in VCO tumors. Within this conceptual framework, vascular promotion emerged as a potential strategy to modulate the tumor vasculature. This was originally established by Wong et al. in pancreatic tumors, where ldCil was used to increase vessel density, verapamil to induce vasodilation and improve blood flow, and gemcitabine as an antitumoral agent [13]. However, these studies were performed exclusively in angiogenic tumors or under VEGF-rich conditions. Overall, according to single-cell data it seems that β_3_ and β_5_ integrin expression is higher in VCO ECs compared with angiogenic lesions, whereas β_1_ integrin expression is lower in VCO ECs [10]. Given this observation and together with our histological analysis indicating the expression of β_1_, β_3_ and β_5_ integrins in VCO and angiogenic lung metastases, we hypothesized a possible vascular promoting effect in these tumors upon ldCil treatment as an inhibitor of these integrins.

Our results demonstrate that ldCil exerts diametrically opposed effects depending on the prevailing vascularization strategy of the tumor. A key finding of our study is that ldCil does not promote but normalizes the co-opted vasculature in the 4T1 lung metastasis model. While conventional antitumoral strategies aim to reduce tumor vascularization, our findings indicate that in VCO the primary challenge is improving the vascular network or impeding the progressive regression collapse due to tumor cell compression [21, 49, 62]. In this context, ldCil enhances vascular function, increasing the available surface area for oxygen exchange and chemotherapy response. This is supported by the increased blood vessel perfusion and concomitant reduction in hypoxia markers expression, indicating that restored vessels are functionally improved. This result highlights that, in non-angiogenic tumors, the therapeutic goal should focus on the restoration of a functional, well-distributed vascular network to alleviate the pro-tumorigenic effects of hypoxia. In contrast, in the RENCA model, where metastases rely on active angiogenesis, ldCil promotes the formation of an expanded but immature vascular network characterized by poor functionality and increased hypoxia, ultimately compromising the efficacy of chemotherapy. These findings reinforce the concept that the use of ldCil cannot be universally applied across tumor types and should instead be tailored according to the HGP of each lesion.

Vascular phenotypes observed in vivo were reproduced in our in vitro endothelial models using HUVECs. Under basal conditions in the absence of VEGF stimulation, ldCil reduced endothelial permeability, improved the organization of intercellular junctions and decreased the number of discontinuous junctions associated with vascular destabilization, supporting its role as a vascular stabilizing agent in quiescent endothelium. In contrast, under VEGF stimulation, ldCil exerts the opposite effect, increasing endothelial permeability, promoting junctional disruption, and enhancing endothelial sprouting, consistent with a pro-angiogenic and destabilizing vascular response. These findings provide a mechanistic explanation for the divergent responses observed in VCO and angiogenic metastases. Nevertheless, a limitation of these in vitro mechanistic studies is the use of HUVECs. Organotropism and vascular remodeling during metastasis are highly context-dependent and HUVECs cell line lack lung-specific endothelial properties. However, HUVECs represent the most widely adopted, robust, and reproducible model to assess general endothelial cell responses in oncology, so our in vitro findings regarding the effect of cilengitide in ECs junctions may be extrapolated to the pulmonary microvascular environment.

We believe that vascular network remodeling constitutes the principal determinant of chemotherapy response in our models, rather than changes in the TIME. This apparent dissociation between vascular and immune remodeling, where changes were limited mainly to differences in CD8^+^ T-lymphocyte infiltration in VCO metastases, may reflect the short duration of our experimental timeline, which likely captures early vascular changes before the immune landscape can be fully reconfigured. Moreover, vascular remodeling was directly associated with alterations that we observed in the number of blood vessels perfused, hypoxia and drug efficacy response to chemotherapy in VCO versus angiogenic lung metastases. The failure of ldCil treatment in the RENCA model is predominantly attributable to impaired perfusion and exacerbated hypoxia, supporting previous reports that vascular promotion strategies in angiogenic tumors may require a vasodilator agent to improve blood flow [13].

Beyond VEGF-mediated pathways, the differential therapeutic responses observed between VCO and angiogenic metastases are likely influenced by differences in ECM composition and vascular maturation states. Our data reveal a markedly higher fibronectin and collagen I deposition in 4T1 metastases compared with RENCA tumors. Excessive fibronectin deposition has recently been associated with endothelial barrier dysfunction [63] and may contribute to the distinct regression state of co-opted blood vessels compared to angiogenic ones, thereby modulating their response to cilengitide. Furthermore, co-opted blood vessels in 4T1 metastases show other hallmarks of active vascular regression, such as a low percentage of pericyte coverage. ldCil appears to reverse this regression by stabilizing the microenvironment and promoting vessel normalization. Conversely, RENCA lesions rely on active, ongoing sprouting angiogenesis where the baseline activation and quiescence states of the ECs are fundamentally different [10], rendering them less susceptible to this specific stabilizing effect. Nevertheless, one of the potential limitations of this study is the difference in growth dynamics between the two metastasis models, requiring the experiments to be carried out over varying timeframes.

In conclusion, our study demonstrates for the first time that the co-opted pulmonary tumor vasculature can be therapeutically remodeled through integrin inhibition with ldCil. Importantly, the effects of this strategy are not universal but depend critically on the underlying HGP of the tumor. While ldCil restores vascular integrity, improves perfusion and enhances chemotherapy efficacy in VCO metastases, it promotes vascular dysfunction and therapy resistance in angiogenic lesions. These findings underscore the need to integrate vascular phenotype stratification into therapeutic decision-making and support the development of vascular context-dependent approaches for the treatment of metastatic lung cancer.

## Supplementary Information

Below is the link to the electronic supplementary material.

Supplementary Material 1.

## Data Availability

No datasets were generated or analysed during the current study.
