# Supplementary material for: Integrin inhibition differentially remodels vascular networks in lung metastases driven by vessel co-option or angiogenesis
Source: Angiogenesis. 2026 Aug 20;29(4):64. doi: 10.1007/s10456-026-10085-1 (PMC13493532; doi:10.1007/s10456-026-10085-1)
Supplement: Supplementary file 1 [file 10456_2026_10085_MOESM1_ESM.docx]

**Integrin inhibition differentially remodels vascular networks in lung metastases driven by vessel co-option or angiogenesis**

Laura Marcos-Zazo^a,b*^, Iván Carrera-Aguado^a,b*^, Jesús Gómez-Escudero^a,b+^, Patricia Berlana-Galán^b,d+^, Irene Torre-Cea^a,b+^, Elena Guerra-Paes^a,b^, Celia Redondo-Gonzalez^a,b^, Juan Sánchez-Mateos^a,b^, Daniel Cáceres-Calle^a,b^, Óscar Maiques^c^, Miguel Pericacho^b,d^, Susana Fraile^e^, Telmo Rodrigues-Teixeira^e^, Carmen García-Macías^e^, Omar García-Sánchez^b,f^, Lorena Benito-Garzón^b,f^, Fernando Sánchez-Juanes^a,b*^ and José M. Muñoz-Félix^a,b*^

a. *Department of Biochemistry and Molecular Biology. University of Salamanca, Salamanca, Spain.*
b. *Institute for Biomedical Research of Salamanca (IBSAL), Salamanca, Spain*
c. *Barts Cancer Institute, Queen Mary University of London, John Vane Science Building, Charterhouse Square, London EC1M 6BQ, UK.*
d. *Department of Physiology and Pharmacology. University of Salamanca, Spain.*
e. *Pathology Unit, Cancer Research Center (CIC-IBMCC), Salamanca, Spain*
f. *Department of Human Anatomy and Histology. University of Salamanca, Salamanca, Spain*

*Correspondence:* [*jmmb@usal.es*](mailto:jmmb@usal.es)*,* [*fsjuanes@usal.es*](mailto:fsjuanes@usal.es)

*^*^Laura Marcos-Zazo and Iván Carrera-Aguado have contributed equally to this manuscript. Fernando Sánchez-Juanes and José M. Muñoz-Félix have contributed equally to this manuscript and share senior authorship.*

*^+^These authors have contributed equally to this manuscript and share second authorship.*

**Supplementary Figures**

***Supplementary Figure 1***

**Supplementary Figure 1. Expression of integrins and ligands in untreated 4T1 and RENCA lung metastases.** **A)** Double immunofluorescence staining of β_3_ or β_5_ integrin/endomucin in 4T1 and RENCA lung metastases. Representative pictures of 4T1 and RENCA metastases treated with PBS stained for β_3_ integrin or β_5_ integrin (red), endomucin (green) and cell nuclei (blue) detection. Co-localization of integrin and endomucin demonstrates that the structures expressing β_3_ and β_5_ integrins are endothelial cells. Scale bar = 50 μm. **B)** Double immunostaining of β_1_ integrin/collagen I and β_1_ integrin/fibronectin in 4T1 and RENCA lung metastases. The left panel displays representative images of 4T1 and RENCA metastases treated with PBS stained for β_1_ integrin (red), collagen I (green) and cell nuclei (blue) detection. Data analysis of collagen I deposition in 4T1 and RENCA lung metastases is also included (*n* = 5 in 4T1 and *n* = 4 in RENCA). Individual values are represented in the graph, and bar represents mean ± SEM. Scale bar = 200 μm. The right panel displays representative images of 4T1 and RENCA metastases treated with PBS stained for β_1_ integrin (red), fibronectin (green) and cell nuclei (blue) detection. Data analysis of fibronectin deposition in 4T1 and RENCA lung metastases is also included (*n* = 5). Individual values are represented in the graph, and the bars represent mean ± SEM. Scale bar = 200 μm. **C)** Collagen I and III deposition detected by picrosirius immunostaining in 4T1 and RENCA. Representative pictures of 4T1 and RENCA tumors treated with PBS stained with picrosirius red for collagen I and III (red) detection. Images were taken with brightfield and polarized light (red selection represents tumor area). Data analysis of collagen I and III levels in the core of the metastasis is also included (*n* = 5). Bar represents mean ± SEM. Scale bar = 200 μm. N.S., non-statistical differences; * *p* ≤ 0.05. Mann-Whitney *U* test (B, C).

***Supplementary Figure 2***

**Supplementary Figure 2. 4T1 and RENCA cell viability, proliferation and migration in response to cilengitide and extracellular matrix analysis in 4T1 and RENCA lung metastases. A)** Cell viability percentage in 4T1 (left panel) and RENCA (right panel) cell lines treated with different concentrations of cilengitide. Individual values are represented in the graph and the bars represent the mean ± SEM. All groups were compared with respect to placebo. **B**) 4T1 and RENCA cells proliferation assessed by Ki67 immunofluorescence after treatment with 1 ng/mL cilengitide. The left panel displays representative pictures of 4T1 cells treated with placebo or 1 ng/mL cilengitide stained for F-actin (green), Ki67 (red) and cell nuclei (blue) detection. Data analysis of 4T1 cells proliferation is also included. Individual values are represented in the graph, and the bars represent the mean ± SEM. Scale bar = 100 μm. The right panel shows representative pictures of RENCA cells treated with placebo or 1 ng/mL cilengitide stained for F-actin (green), Ki67 (red) and cell nuclei (blue) detection. Data analysis of RENCA cells proliferation is also included. Individual values are represented in the graph, and the bars represent the mean ± SEM. Scale bar = 100 μm. **C)** Migration capacity of 4T1 (left panel) and RENCA (right panel) cells lines treated with 1 ng/mL cilengitide. The left panel shows representative pictures of the wound healing assays performed in 4T1 cells treated with 1 ng/mL. Data analysis of the wound area percentage is included. In the graph, each point represents mean ± SEM. All groups were compared at every time point. The right panel shows representative pictures of the wound healing assays performed in RENCA cells treated with 1 ng/mL cilengitide. Data analysis of the wound area percentage is included. In the graph, each point represents the mean ± SEM. All groups were compared at every time point. **D**) 4T1 (upper panel) and RENCA (lower panel) cells adhesion to collagen I or fibronectin in presence of different concentrations of cilengitide. Individual values are represented in the graph, and the bars represent the mean ± SEM. All groups were compared with respect to placebo. **E**) 4T1 (left panel) and RENCA (right panel) cells adhesion to collagen I or fibronectin in presence of 2 ng/mL cilengitide in combination or not with the α_5_β_1_ inhibitor isoDGR. Individual values are represented in the graph, and the bar represents mean ± SEM. N.S., non-statistical differences; * *p* ≤ 0.05; ** *p* ≤ 0.01; *** *p* ≤ 0.001; **** *p* ≤ 0.0001. Kruskal-Wallis test, post hoc Dunn’s test (A, D, E); Mann-Whitney *U* test (B); two-way ANOVA, post hoc Tukey’s test (C).

***Supplementary Figure 3***

**Supplementary Figure 3. Effect of ldCil on kidney tissue of tumor-free mice. A)** H&E representative pictures of renal cortex and renal medulla from healthy mice treated with placebo (left panel) or ldCil (right panel). ldCil treatment does not modify glomeruli morphology or tubular interstitium ultrastructure. Yellow lines delineate glomeruli. Scale bar = 200 μm. **B)** Endomucin (red) staining representative pictures of renal cortex from healthy mice treated with placebo or ldCil. ldCil treatment does not produce changes in glomerular and peritubular blood vessel area. White lines indicate blood capillaries which were considered to correspond to glomeruli. Data analysis of glomerular capillary area and peritubular capillary area are also included (*n* = 7). Individual values are represented in the graph, and the bars represent the mean ± SEM. Scale bar = 200 μm. N.S., non-statistical differences. Student’s *t* test (B).

***Supplementary Figure 4***

**Supplementary Figure 4. Effect of sorafenib and sorafenib combined with cilengitide on 4T1 and RENCA lung metastases. A)** Number of metastases, tumor area and mean tumor size in 4T1 lung metastases treated with 10 mg/kg sorafenib and 10 mg/kg sorafenib combined with 50 μg/kg ldCil. Representative H&E staining pictures of mouse lung lobes bearing 4T1 metastases treated with sorafenib in monotherapy or in combination with 50 μg/kg cilengitide. Background of the lobes of H&E staining images has been removed using PowerPoint in order to clarify the observation of the lung metastases. Data analysis of the number of metastases, tumor area and mean tumor size are also included (*n* = 6 in sorafenib and *n* = 7 in sorafenib + ldCil). Individual values are represented in the graph, and the bars represent the mean ± SEM. Scale bar = 1 mm (gross lung) and 200 μm (zoomed area). **B)** Endomucin/PDGFRβ staining in 4T1 lung metastases treated with 10 mg/kg sorafenib and 10 mg/kg sorafenib combined with 50 μg/kg ldCil. The upper panel shows representative pictures of 4T1 metastases stained for endomucin (green) and PDGFRβ (red). White arrows indicate blood vessels covered with pericytes. Data analysis of the number of blood vessels per area and pericyte coverage is also included (*n* = 6 in sorafenib and *n* = 7 in sorafenib with ldCil). Blood vessel density has been represented multiplying the blood vessel/tumor area ratio by the correction factor 10^6^. Individual values are represented in the graph, and the bars represent the mean ± SEM. Scale bar = 50 μm. N.S., non-statistical differences. **C)** Number of metastases, tumor area, mean tumor size and perimeter/area ratio in RENCA lung metastases after 10 mg/kg sorafenib and 10 mg/kg sorafenib combined with 50 μg/kg ldCil treatments. Representative H&E staining pictures of a mouse lung lobe bearing RENCA metastases treated with sorafenib in monotherapy or in combination with 50 μg/kg cilengitide. Background of the lobes of H&E staining images has been removed using PowerPoint in order to clarify the observation of the lung metastases. Data analysis of the number of metastases, tumor area, mean tumor size and perimeter/area ratio are also included (*n* = 6 in sorafenib and *n* = 7 in sorafenib with ldCil). Individual values are represented in the graph, and the bars represent the mean ± SEM. Scale bar = 1 mm (gross lung) and 200 μm (zoomed area). **D)** Podoplanin/endomucin staining in RENCA lung metastases treated with 10 mg/kg sorafenib and 10 mg/kg sorafenib combined with 50 μg/kg ldCil. Representative pictures of RENCA metastases stained for podoplanin (green) and endomucin (red) are shown. Data analysis of the HGP distribution, blood vessel density and *Epithelial Loss Area* are also included (*n* = 5 in sorafenib and *n* = 8 in sorafenib with ldCil). Blood vessel density has been represented by multiplying the blood vessel/tumor area ratio by the correction factor 10^6^. Individual values are represented in the graph, and the bars represent the mean ± SEM. Scale bar = 200 μm. N.S., non-statistical differences; * *p* ≤ 0.05; ** *p* ≤ 0.01. Student’s *t* test (A, C: number of metastases, tumor area and mean tumor size; B, D: blood vessels/tumor area); Mann-Whitney *U* test (C: perimeter/area ratio; B, D: blood vessel coverage); Fisher's exact test (D: HGP distribution).

***Supplementary Figure 5***

**Supplementary Figure 5. Effect of cilengitide on chemotherapy efficacy in 4T1 and RENCA lung metastases.**

**A)** Cell viability percentage in 4T1 (left panel) and RENCA (right panel) cell lines after treatment with different doses of paclitaxel in presence or absence of 1 ng/mL cilengitide. Each point represents the mean ± SEM. Statistical analysis was employed to elucidate differences in cell viability between the presence and the absence of cilengitide at the same chemotherapy dose. **B)** Number of metastases, tumor area and mean tumor size after 5 mg/kg carboplatin (Carbo^5^) and ldCil with Carbo^5^ treatments in 4T1 experimental lung metastases. The upper panel shows representative images of a mouse lung lobe bearing 4T1 metastases treated with Carbo^5^ or Carbo^5^ combined with 50 μg/kg cilengitide stained with H&E. The background of the H&E staining images has been removed using PowerPoint in order to clarify the observation of the lung metastases. The lower panel shows data analysis of the number of metastases, tumor area and mean tumor size (*n* = 5). Individual values are represented in the graph, and the bar represents mean ± SEM. Scale bar = 1 mm (gross lung) and 200 μm (zoomed area). **C)** Cell viability percentage in 4T1 cells following treatment with different doses of carboplatin in presence or absence of 1 ng/mL of cilengitide. Each point represents the mean ± SEM. Statistical analysis was employed to elucidate differences between the presence and the absence of cilengitide at the same chemotherapy dose. N.S., non-statistical differences; * *p* ≤ 0.05; ** *p* ≤ 0.01; *** *p* ≤ 0.001; **** *p* ≤ 0.0001. Two-way ANOVA, post hoc Tukey’s test (A, C); Student's *t* test (B).

**Supplementary Tables**

**Supplementary Table 1. Primary antibodies used in immunohistochemistry, cell immunofluorescence and Western Blot.**

| Target | Primary antibody |
| --- | --- |
| β_1_ integrin | Immuno: Santa Cruz Biotechnology Cat# sc-9970, RRID:AB_ 627004  Western Blot: Santa Cruz Biotechnology Cat# sc-374429, RRID:AB_11012020 |
| β_3_ integrin | Santa Cruz Biotechnology Cat# sc-365679, RRID:AB_10844835 |
| β_5_ integrin | Santa Cruz Biotechnology Cat# sc-398214, RRID:AB_3713205 |
| GLUT-1 | Thermo Fisher Scientific Cat# MA5-31960, RRID:AB_2809254 |
| CAIX | Abcam Cat# ab270401, RRID:AB_3665691 |
| CD8 | Abcam Cat# ab217344, RRID:AB_2890649 |
| Arginase-1 | Cell Signaling Technology Cat# 93668, RRID:AB_2800207 |
| FOXP3 | Cell Signaling Technology Cat# 12653, RRID:AB_2797979 |
| Granzyme B | Proteintech Cat# 13588-1-AP, RRID:AB_2114429 |
| CD11b | Abcam Cat# ab133357, RRID:AB_2650514 |

**Supplementary Table 2. Antigen retrieval solution used in immunofluorescence and immunohistochemistry.**

| Antigen retrieval solution | Staining |
| --- | --- |
| Sodium citrate buffer 10 mM pH 6 with 0.05 % Tween-20 and heating in the microwave | Endomucin/Podoplanin |
|  | Endomucin/PDGFRβ |
|  | Endomucin/Desmin |
|  | Endomucin/β_3_ integrin |
|  | Endomucin/β_5_ integrin |
|  | Endomucin/Ki67 |
|  | GLUT-1 |
|  | CAIX |
|  | Arginase-1 |
| Proteinase-K (Sigma-Aldrich) diluted 1/25 in Tris 50mM-EDTA 1 mM (pH 8) (Fisher Bioreagents) (10 minutes at 50 ºC) | β_1_ integrin/Fibronectin |
|  | β_1_ integrin/Collagen I |
|  | β_3_ integrin |
|  | β_5_ integrin |
| TRIS 50 mM-EDTA 1 mM (pH 9) (Fisher Bioreagents) (10 minutes at maximum power and 10 minutes at medium power) | CD8 |
|  | CD11b |
|  | Granzyme B |
|  | FOXP3 |

**Supplementary Table 3. Primary and secondary antibodies used in immunofluorescence staining.**

|  | | Primary antibody | Secondary antibody |
| --- | --- | --- | --- |
| Endomucin/podoplanin staining | Endomucin | Santa Cruz Biotechnology Cat# sc-65495, RRID:AB_2100037 | Sigma-Aldrich Cat# SAB4600085, RRID:AB_3492074 |
|  | Podoplanin | Thermo Fisher Scientific Cat# 14-5381-85, RRID:AB_1210507 | Abcam Cat# ab180063, RRID:AB_2891031 |
| Endomucin/PDGFRβ  staining | Endomucin | Santa Cruz Biotechnology Cat# sc-65495, RRID:AB_2100037 | Thermo Fisher Scientific Cat# A-11006, RRID:AB_2534074 |
|  | PDGFRβ | Abcam Cat# ab69506, RRID:AB_3719920 | Thermo Fisher Scientific Cat# A-21124, RRID:AB_2535766 |
| Endomucin/Desmin staining | Endomucin | Santa Cruz Biotechnology Cat# sc-65495, RRID:AB_2100037 | Sigma-Aldrich Cat# SAB4600085, RRID:AB_3492074 |
|  | Desmin | Proteintech Cat# 16520-1-AP, RRID:AB_2292918 | Thermo Fisher Scientific Cat# A-11034, RRID:AB_2576217 |
| Endomucin/Ki67 staining | Endomucin | Santa Cruz Biotechnology Cat# sc-65495, RRID:AB_2100037 | Sigma-Aldrich Cat# SAB4600085, RRID:AB_3492074 |
|  | Ki67 | Proteintech Cat# 28074-1-AP, RRID:AB_2918145 | Thermo Fisher Scientific Cat# A-11034, RRID:AB_2576217 |
| β_1_ integrin/ fibronectin staining | β_1_ integrin | Santa Cruz Biotechnology Cat# sc-9970, RRID:AB_627004 | Thermo Fisher Scientific Cat# A-21124, RRID:AB_2535766 |
|  | Fibronectin | Abcam Cat# ab23750, RRID:AB_447655 | Thermo Fisher Scientific Cat# A-11008, RRID:AB_143165 |
| β_1_ integrin/ collagen I staining | β_1_ integrin | Santa Cruz Biotechnology Cat# sc-9970, RRID:AB_627004 | Thermo Fisher Scientific Cat# A-21124, RRID:AB_2535766 |
|  | Collagen I | Abcam Cat# ab21286, RRID:AB_446161 | Thermo Fisher Scientific Cat# A-11008, RRID:AB_143165 |
| β_3_ integrin/ endomucin | β_3_ integrin | Santa Cruz Biotechnology Cat# sc-365679, RRID:AB_10844835 | Thermo Fisher Scientific Cat# A-21124, RRID:AB_2535766 |
|  | Endomucin | Santa Cruz Biotechnology Cat# sc-65495, RRID:AB_2100037 | Thermo Fisher Scientific Cat# A-11006, RRID:AB_2534074 |
| β_5_ integrin/ endomucin | β_5_ integrin | Santa Cruz Biotechnology Cat# sc-398214, RRID:AB_3713205 | Thermo Fisher Scientific Cat# A-21124, RRID:AB_2535766 |
|  | Endomucin | Santa Cruz Biotechnology Cat# sc-65495, RRID:AB_2100037 | Thermo Fisher Scientific Cat# A-11006, RRID:AB_2534074 |

**Supplementary material and methods**

***Tumor cells viability assay***

For 4T1 and RENCA cell viability assays, we seeded 5·10^3^ cells per well (100 μL/well) into a 96-well dish. After 2 days growing at 37 ºC and 5 % CO_2_, we added 100 μL of the corresponding treatment: 0.1 ng/mL, 1 ng/mL, 10 ng/mL, 100 ng/mL, 1 μg/mL and 10 μg/mL of cilengitide (Selleckchem) or 1 ng/mL of cilengitide in presence or absence of different concentrations of paclitaxel (MedChem) (0.1 μM, 1 μM, 10 μM and 100 μM) in 4T1 and RENCA cell line and carboplatin (MedChem) (100 ng/mL, 1 μg/mL, 10 μg/mL and 100 μg/mL) in 4T1 cells. Subsequently, the same protocol described in the materials and methods section was applied.

***Tumor cells proliferation assay***

Immunocytochemistry for Ki67 detection was performed in 4T1 and RENCA cell lines to evaluate their proliferation capacity under ldCil treatment. 5·10^3^ cells were seeded on µ-Slide 15 Well 3D ibidi slides (IBIDI) and cultured until they reached confluence. Subsequently, the same protocol described in cell immunofluorescence in the materials and methods section was applied. In this case, the primary antibody anti-Ki67 Proteintech Cat# 28074-1-AP, RRID:AB_2918145 and the secondary antibody Thermo Fisher Scientific Cat# A-11036, RRID:AB_10563566 were applied. Images of the samples were taken with Zeiss Axioplan fluorescence microscope and percentage of proliferation cells were calculated by dividing the number of Ki67-positive nuclei by the total number of cells.

***Tumor cells migration assay***

4T1 and RENCA cells were cultured to evaluate the capacity to migrate in presence of 1 ng/mL. For this assay, cells were seeded in 6 well-dishes until 95 % confluence. In this moment, cells were treated with the corresponding cilengitide concentration and 15 minutes later, a wound was made in each well with a new 0.2-mL pipette tip in order to check cell migration to seal it. Images of the wells were taken 0, 24 and 48 hours post-wound with an Invitrogen™ EVOS™ XL Core Imaging System. Subsequently, images were analysed using the Wound Healing Size Tool plugin from Fiji/ImageJ software (1.54f version on Windows) in order to obtain the wound area percentage.

***Cell adhesion assay***

Cell adhesion assays were performed to check whether cilengitide inhibited the adhesion of 4T1 and RENCA cells to a coating matrix of fibronectin or collagen I. Wells of 96-well plates were coated with a 50 μL solution of a matrix consisting of 5 μg/mL fibronectin (Sigma-Aldrich) or 5 μg/mL collagen I (Sigma-Aldrich) diluted in Bovine Gelatine 0.1 % (Sigma-Aldrich). Dishes were incubated for 2 hours at room temperature until matrix gelation, and the remnant matrix was removed before washing each well with PBS (Sigma-Aldrich). Subsequently, the blocking solution (cell culture medium) was added to each well and dishes were incubated for 30 minutes at 37 ºC. After blocking, the medium was discarded. Immediately prior to seeding the cells, 50 μL of a solution with the corresponding cilengitide (Selleckchem) treatment or the α_5_β_1_ integrin inhibitor based on the RGD sequence c(phg-isoDGR-(NMe)k)TFA (isoDGR) (MedChem) treatment was added to each well at twice the final concentration. Afterwards, 4T1 and RENCA cell lines were trypsinized, counted in a Neubauer counting chamber (Thermo Fisher Scientific) and seeded in the wells containing the treatment solution (50 μL and 5·10^3^ cells per well). Hence, the final volume of each well resulted in the intended concentration of each treatment (0.5 ng/mL, 1 ng/mL and 2 ng/mL cilengitide or 1 μg/mL isoDGR). Then, dishes were incubated for 6 hours at 37 ºC to allow cell adhesion to the coating matrix. After the incubation period, the medium was removed, the wells were washed twice with PBS and the remaining cells were fixed with 100 % methanol (Sigma-Aldrich) for 10 minutes at room temperature. After fixation, wells were washed three times with PBS and cells were stained with a solution of 0.5 % crystal violet (Sigma-Aldrich) diluted in ethanol 20 % at room temperature. The excess dye was removed by three 1-minute immersions in distilled water and dishes were left to dry overnight at room temperature. The next day, images of the plates were taken using an Invitrogen™ EVOS™ XL Core Imaging System. In order to quantify cell adhesion, 100 μL of 100 % methanol were added to each well and dishes were kept in agitation for 15 minutes at room temperature. Finally, absorbance was measured at 595 nm using a Thermo Scientific™ Multiskan™ FC Microplate Photometer in order to obtain the percentage of cell adhesion.

***Immunofluorescence***

We carried out several immunofluorescence stainings: β_1_ integrin/fibronectin, β_1_ integrin/collagen I and β_3_ or β_5_ integrin/endomucin. The protocol described in the materials and methods section was carried out and the corresponding primary antibodies were applied (Supplementary Table 3).

***Picrosirius red staining***

Picrosurius red staining was used for collagen types I and III deposition analysis. Following the manufacturer’s protocol (ScyTek Laboratories), tissue sections were first deparaffinized and rehydrated using Clear SX85 (Casa Álvarez) and a graded ethanol series (100 %, 80 %, 70 %, and 50 %). After rinsing with distilled water, the samples were incubated in Picrosirius Red solution (ScyTek Laboratories) for 60 minutes, then washed twice in 0.5 % acetic acid. Finally, the tissues were dehydrated in 100 % ethanol for 4 minutes, cleared with Clear SX85, and mounted using DPX mounting medium (Casa Álvarez). Lung sections were scanned using a ZEISS Axioscan 7 microscope equipped with polarized light filters. Images were analyzed using QuPath software (version 0.5.1) with custom scripts that automatically detected collagen I and III corresponding to tumor tissue and tumor free-lung (tumor adjacent parenchyma).

***Evaluation of the effect of ldCil on healthy tissue***

An *in vivo* experiment was performed using healthy *wild type* C57BL/6 mice from an in-house colony to evaluate ldCil effect on highly vascularized non-tumoral tissue, such as the kidneys. Mice were split into two treatment groups: placebo (*n =* 7) or ldCil (*n =* 7). Placebo mice were treated intraperitoneally with 100 μL PBS, whereas ldCil group was treated intraperitoneally with cilengitide (50 μg/kg). A total of three doses were administered, at 3-day intervals. Three days after the last treatment, mice were euthanized. Kidneys were collected, fixed in 3.7 % PFA (Casa Álvarez) for 24 hours and incubated in 70 % ethanol (Casa Álvarez) for 24 hours. Subsequently, paraffin embedding was performed, and 3 μm sections were obtained and mounted on glass slides to perform H&E staining and endomucin staining according to the protocols described in the materials and methods section.

***Analysis of glomerular and peritubular vessels analysis in healthy mice kidney***

Quantification of glomerular and peritubular vascular area in kidney sections was performed using Fiji/ImageJ software (1.54f version on Windows). Capillary structures were defined and delineated using the Threshold tool, with threshold levels adjusted to optimize vascular segmentation prior to quantification. Then, total vascular area and total tubular vascular area were measured, and glomerular vascular area was calculated by subtracting tubular vascular area from total vascular area.

***Evaluation of the VEGF-dependent effects of ldCil through sorafenib-mediated VEGFR2 inhibition***

An *in vivo* experiment was performed using the anti-angiogenic drug sorafenib, a tyrosine kinase inhibitor that blocks VEGFR2 and PDGFRβ activity [1]. We intended to replicate the effect of ldCil in the VCO 4T1 model in presence of an anti-angiogenic drug to demonstrate its VEGF-independent mechanism. Six-week-old BALB/c female mice were injected intravenously into the tail vein with 2·10^5^ 4T1 cells and split into two groups: sorafenib (*n =* 6) and sorafenib in combination with ldCil (*n =* 7) [2]. Mice were treated intraperitoneally with 10 mg/kg sorafenib (MedChem) diluted in corn oil (Sigma-Aldrich) or 10 mg/kg sorafenib combined with 50 μg/kg cilengitide, following the treatment schedule described in the materials and methods section. Lung tissues were processed for H&E and endomucin/PDGFRβ stainings, procedures that are described in the manuscript. The sorafenib dose was chosen based on published research by Lozano et al., 2019 [3].

***Evaluation of the effects of ldCil in VCO growth as resistance to Sorafenib in the RENCA model***

Given that angiogenic tumors subjected to AAT, such as Sunitinib [4], Bevacizumab [5], or Sorafenib [6] develop VCO, an *in vivo* experiment was performed to evaluate whether ldCil is capable of reducing this VCO-driven growth in sorafenib-treated RENCA metastases.

Six-week old BALB/c female mice (Charles River) were injected intravenously into the tail vein with 2·10^5^ RENCA cells [2]. Then, mice were treated intraperitoneally with 10 mg/kg sorafenib (MedChem) diluted in corn oil (Sigma-Aldrich) or 10 mg/kg sorafenib combined with 50 μg/kg cilengitide, following the treatment schedule described in the materials and methods section. Lung tissues were processed for H&E and endomucin/podoplanin staining, procedures that are described in the manuscript. The sorafenib dose was chosen based on published research by Lozano et al., 2019 [3].

***β_1_ integrin, collagen I and fibronectin analysis***

Immunofluorescence images of β_1_ integrin/fibronectin and β_1_ integrin/collagen I from 4T1 and RENCA metastases treated with PBS or ldCil were analysed using the ImageJ/Fiji software (version 1.54f, Windows) to determine the percentage of fibronectin or collagen I. Seven tumors per mouse were randomly selected to perform the analysis and the mean of each mouse was calculated. Fibronectin or collagen I-positive areas were identified using the Color Threshold tool, while the total tumor area was calculated by manually delineating the metastases. Area measurements are reported in pixels.

***Evaluation of carboplatin sensitization in 4T1 lung metastases using ldCil***

An *in vivo* experiment was carried out to evaluate the benefits of another chemotherapy regimen when using ldCil in the 4T1 model. Animals bearing 4T1 lung metastases were treated with carboplatin (MedChem) (Carbo^5^) diluted in PBS or Carbo^5^ with 50 μg/kg cilengitide at days 4, 7 and 10 post-injection following the schedule treatment described previously. Mice were sacrificed on day 13.

**Supplementary references**

1.	Wilhelm S, Carter C, Lynch M, et al (2006) Discovery and development of sorafenib: a multikinase inhibitor for treating cancer. Nat Rev Drug Discov 5:835–844. https://doi.org/10.1038/nrd2130

2.	Cuypers A, Teuwen L-A, Bridgeman VL, et al (2022) Generation of vessel co-option lung metastases mouse models for single-cell isolation of metastases-derived cells and endothelial cells. STAR Protoc 3:101691. https://doi.org/10.1016/j.xpro.2022.101691

3.	Lozano E, Macias RIR, Monte MJ, et al (2019) Causes of hOCT1‐Dependent Cholangiocarcinoma Resistance to Sorafenib and Sensitization by Tumor‐Selective Gene Therapy. Hepatology 70:1246–1261. https://doi.org/10.1002/hep.30656

4.	Teuwen L-A, De Rooij LPMH, Cuypers A, et al (2021) Tumor vessel co-option probed by single-cell analysis. Cell Rep 35:109253. https://doi.org/10.1016/j.celrep.2021.109253

5.	Frentzas S, Simoneau E, Bridgeman VL, et al (2016) Vessel co-option mediates resistance to anti-angiogenic therapy in liver metastases. Nat Med 22:1294–1302. https://doi.org/10.1038/nm.4197

6.	Kuczynski EA, Yin M, Bar-Zion A, et al (2016) Co-option of Liver Vessels and Not Sprouting Angiogenesis Drives Acquired Sorafenib Resistance in Hepatocellular Carcinoma. J Natl Cancer Inst 108:djw030. https://doi.org/10.1093/jnci/djw030
